# Clathrins Are Involved in the Endocytosis of Host Cytosol in the Malaria Parasite

**DOI:** 10.1111/tra.70025

**Published:** 2025-12-26

**Authors:** Jun Miao, Amuza Byaruhanga Lucky, Gang Ning, Xian Xia, Xiaoying Liang, Faiza Siddiqui, Hui Min, Chengqi Wang, Xiaolian Li, Z. Hong Zhou, Liwang Cui

**Affiliations:** ^1^ Department of Internal Medicine Morsani College of Medicine, University of South Florida Tampa Florida USA; ^2^ Center for Global Health and Infectious Diseases Research College of Public Health, University of South Florida Tampa Florida USA; ^3^ Huck Institute of Life Sciences, the Pennsylvania State University University Park Pennsylvania USA; ^4^ Department of Microbiology Immunology & Molecular Genetics, University of California Los Angeles California USA

**Keywords:** adaptor protein, clathrin, cytostome, endocytosis, hemoglobin uptake, malaria

## Abstract

In eukaryotic cells, clathrins interact with the adaptor protein (AP) complex‐2 (AP‐2) to facilitate endocytosis and AP‐1 to mediate secretion and trafficking between the endosome and Golgi. In *Plasmodium falciparum*, recent studies revealed that the Kelch domain‐containing protein 13 and AP‐2 participate in hemoglobin uptake via cytostomes. However, clathrins appear not to be involved in this process because they primarily associate with AP‐1. To investigate the roles of clathrins in *P. falciparum*, we characterized the clathrin heavy chain (*Pf*CHC), the clathrin light chain (*Pf*CLC), and the AP‐1 γ subunit (*Pf*AP‐1 γ). Extensive interactome analyses confirmed the major association of clathrins with AP‐1 components alongside proteins involved in cytostome formation. Live‐cell imaging and protein colocalization studies showed that *Pf*CHC, *Pf*CLC, and *Pf*AP‐1 γ are localized in the parasite cytoplasm, predominantly at the parasite periphery and near the *cis*‐Golgi. Ultrastructural studies using ascorbate peroxidase 2‐based electron microscopy confirmed their presence at coated vesicle‐like structures at the parasite periphery and, unexpectedly, at the collars of cytostomes. Knockdown of *Pf*CHC led to the formation of abnormally long cytostome tubes and impaired hemoglobin uptake, suggesting that clathrins are involved in cytostome processes in *P. falciparum*.

## Graphical abstract

This study characterized clathrin function in hemoglobin uptake from host red blood cells via the malaria parasite's specific endocytosis machinery, cytostome. Clathrin heavy chain, clathrin light chain, and AP‐1 γ subunit were found associated with certain proteins known to be involved in cytostome function, and they were localized at the collars of cytostomes. Knockdown of *Pf*CHC resulted in dysfunctional, long tube‐like cytostomes and reduced hemoglobin uptake, highlighting the important role of clathrins in the parasite's hemoglobin uptake and digestion processes.

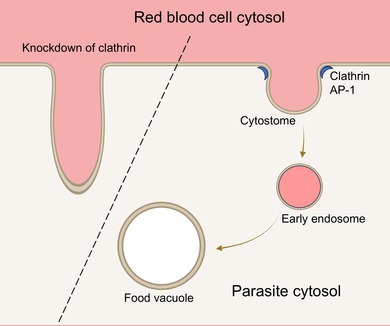

## 1 Introduction

Clathrin‐coated vesicles (CCVs) in eukaryotes are well‐described [1, 2, 3, 4, 5, 6]. Clathrins form a three‐legged structure termed triskelion, which is composed of three identical clathrin heavy chains (CHCs), each bound to a single clathrin light chain (CLC) [2]. Triskelia are the assembly units of the polygonal lattice that make up the basket‐like clathrin‐coated vesicles [2, 7]. Clathrins are associated with the heterotetrameric adaptor protein (AP) complexes that link clathrin to CCV's cargoes [1, 2, 3, 4, 5, 6]. Each AP complex contains two large subunits (α/γ/δ/ε/ζ and β1‐5) of ~100 kDa, a medium subunit (μ1–5) of ~50 kDa, and a small subunit (σ1–5) of ~20 kDa. APs are classified into five types (AP1–5). AP‐1 is involved in traffic from the *trans*‐Golgi network to the endosome or polarized plasma membrane for secretion, while AP‐2 is located at the plasma membrane for endocytosis [2, 7, 8, 9, 10, 11]. While AP‐3 also plays a role in the traffic between the Golgi and endosomes, AP‐4 and AP‐5 are not associated with coated vesicles [2, 12]. Different sorting signals present in cargo molecules are recognized by different subunits of AP complexes [2, 12].

Of the five malaria species infecting humans, *Plasmodium falciparum* causes the most severe malaria and is responsible for nearly half a million deaths annually [13]. The *P. falciparum* genome encodes most components of the clathrin complexes [14, 15]. Recent studies found that *Pf*CHC, *Pf*AP‐1 μ1, and *Pf*AP‐2 μ2 were localized in the cytosol of the parasite, and *Pf*AP‐2 μ2 is essential for schizont maturation [16, 17, 18]. Yet, the significance of CCVs in malaria parasites has not been investigated.

Apicomplexan parasites take up the host cytoplasm by endocytosis via a structure called micropore, which comprises parasite plasma membrane invagination surrounded by electron‐dense rings at its collar [19, 20, 21]. Type I micropore occurs in openings of the inner membrane complex of *Plasmodium* sporozoite and *T*. *gondii*. In *Plasmodium*, the cytostome (also called type II micropore) has an additional parasitophorous vacuolar membrane invagination, but without the restriction of the inner membrane complex, and is specific for the uptake of host cell hemoglobin [19]. In *P. falciparum*, Kelch domain‐containing protein 13 (PfK13), the key determinant of artemisinin resistance [17, 18, 21, 22, 23, 24], and *Pf*AP‐2 complex‐associated proteins were localized at the cytostome collar [17, 18, 22, 24, 25]. Similar localization patterns of K13 and AP‐2 subunits were confirmed in *T*. *gondii* [26, 27]. These findings suggest the involvement of K13 and AP‐2 complex in endocytosis in both *P. falciparum* and *T. gondii*. Consistently, knockdown (KD) of K13 and its associated proteins in both species disturbed the formation of the cytostome or micropore and affected the uptake of hemoglobin or host cell materials [18, 24, 26, 27]. Unexpectedly, clathrins were not associated with the *Pf*AP‐2 complex, suggesting that they do not play a major role in endocytosis. Instead, *Pf*CHC was mainly associated with the AP‐1 complex in *P. falciparum* [17, 18]. Therefore, clathrins may play parasite‐specific roles in the malaria parasite.

In this study, the unique features of clathrins and their predominant association with the AP‐1 complex in *P. falciparum* prompted us to characterize their functions in vesicle transport. Through extensive interactome, localization, and gene manipulation studies, we present evidence that clathrins and AP‐1 are involved in endocytosis of hemoglobin via mediating cytostome processes.

## 2 Results

### 2.1 *Plasmodium falciparum* Encodes Conserved Clathrins and AP‐1 Complex

While the *Pf*CHC (PF3D7_1219100) has a conserved domain structure organization with ~40% identity to CHCs in model eukaryotic organisms, it contains five Asn‐rich insertions longer than 28 aa, accounting for an overall increase of protein size by > 300 amino acids (aa) (Figure S1A, S1B). Despite these insertions, the region predicted to interact with the CLC and residues (Q89, F91, K96, and K98) critical for β‐arrestin and β‐adaptin binding in the N‐terminal β‐propeller domain in vertebrate CHCs [28, 29, 30, 31] is perfectly conserved in *Pf*CHC. *Pf*CLC (PF3D7_1435500) is also conserved in domain organization but only has ~20% identity to vertebrate CLCs. In the consensus region, the acidic patch (DEG vs. EED in human CLC) and residues (W153 and I175 in *Pf*CLC vs. W105 and W127 in human CLC) critical for binding CHC [32, 33] are relatively conserved (Figure S1C, S1D, Figure S2). *Pf*AP‐1 γ subunit (PF3D7_1455500) has ~30% identity to vertebrate AP‐1 γ1s with a 99‐aa insertion in the trunk domain and a longer unstructured linker (Figure S1E, S1F). The other AP‐1 subunits, *Pf*AP‐1 β1, μ1, and σ1, display higher identities (~48%, ~60%, and ~57%) to their respective vertebrate counterparts, respectively (Figure S1G–L). *Pf*CHC, *Pf*CLC, and *Pf*AP‐1 subunits show the highest levels of sequence identity to their respective orthologs from *T. gondii* (Figure S1). These results indicate that *P. falciparum* clathrins and AP‐1 subunits harbor both conserved and diverse domain structures.

### 2.2 Affinity Pulldowns Identify Proteins Involved in Cytostome Formation

Previous reports showed that *Pf*CHC was mainly associated with AP‐1 subunits by proximity labeling (BioID) and affinity purification [17, 18]. To confirm these findings, we tagged the endogenous *Pf*CHC, *Pf*CLC, and *Pf*AP‐1 γ with GFP using a single‐crossover recombination strategy (Figures S3A, S3B). Western blots with anti‐GFP antibodies revealed ~260, ~60, and ~150 kDa bands, consistent with the molecular weights predicted from the GFP‐fused *Pf*CHC, *Pf*CLC, and *Pf*AP‐1 γ, respectively (Figure S3C–E). The expression levels of these proteins were increased during the intraerythrocytic developmental cycle (Figure S3C–E).

We first precipitated proteins from trophozoites of the *Pf*CHC::GFP parasite line using GFP‐trap agarose. Using liquid chromatography and tandem mass spectrometry, we identified 87 proteins using stringent parameters by Significance Analysis of INTeractome (SAINT) analysis (a threshold of probability above 90% and false discovery rate below 2.5%) [34] (Table S1). *Pf*CHC, *Pf*CLC, and AP‐1 subunits were identified at high abundance, confirming that *Pf*CHC is principally associated with AP‐1 (Table 1). Comparing this interactome result with the 40 and 63 *Pf*CHC‐associated proteins identified previously by BioID and anti‐GFP magnetic beads, respectively [17, 18], we identified nine shared proteins, including *Pf*CHC, *Pf*CLC, AP‐1 subunits, Sortilin (an escort protein for trafficking) [35, 36], and a putative AP‐4 complex accessory subunit Tepsin (Table 1, Table S1). The identification of Tepsin suggests that clathrins may be associated with the AP‐4 complex [18].

**TABLE 1:** Important proteins identified by GFP‐trap from *Pf*CHC, *Pf*CLC, and *Pf*AP‐1 γ IPs.

Gene ID	Gene name	Protein length	Control IP			*Pf*CHC IP		P (%)	FDR (%)	*Pf*CLC IP			P (%)	FDR (%)	*Pf*AP‐1 γ IP			P (%)	FDR (%)
			R1	R2	R3	R1	R2			R1	R2	R3			R1	R2	R3		
PF3D7_1219100	CHC	1997	3	0	0	483	591	100	0	1826	1888	1956	100	0	117	112	103	100	0
PF3D7_1435500	CLC	254	0	0	0	36	42	100	0	178	174	164	100	0	4	9	8	100	0.05
PF3D7_1455500	AP‐1 γ	1081	0	0	0	66	49	100	0	118	118	115	100	0	462	449	432	100	0
PF3D7_0528100	AP‐1/2 β	929	0	0	0	60	62	100	0	87	104	101	100	0	35	31	37	100	0
PF3D7_1311400	AP‐1 μ1	437	0	0	0	31	27	100	0	26	33	29	100	0	18	14	12	100	0
PF3D7_1118100	AP‐1 σ1	155	0	0	0	4	8	98	0.19	20	21	23	100	0	8	8	7	100	0
PF3D7_1451800	SORTLR	895	0	0	0	48	26	100	0	25	22	31	100	0	0	0	0	0	0
PF3D7_1411300	DnaJ	568	0	0	0	9	10	100	0	62	68	62	100	0	3	5	4	99	0.14
PF3D7_1421000	DIX	177	0	0	0	3	9	97	0.6	61	62	56	100	0	2	0	0	29	16.4
PF3D7_1459600	Tepsin	801	0	0	0	32	67	100	0	85	85	89	100	0	4	4	4	100	0.04
PF3D7_0904100	AP‐4 ε	1388	0	0	0	0	0	0	0	4	12	6	100	0.02	0	0	0	0	0
PF3D7_0730200	AP‐4 β	858	0	0	0	0	0	0	0	3	7	5	100	0.15	0	0	0	0	0
PF3D7_0423100	AP‐4 σ	146	0	0	0	0	0	0	0	3	3	2	96	0.83	1	0	1	0	76.2
PF3D7_1119500	AP‐4 μ4	436	0	0	0	0	0	0	0	1	2	2	88	2.7	1	0	1	0	0
PF3D7_1218300	AP‐2 μ2	621	0	0	0	0	0	0	0	6	8	3	99	0.2	4	2	3	98	0.47
PF3D7_0617100	AP‐2 α	1236	0	0	0	0	0	0	0	3	4	6	99	0.12	0	0	0	0	0
PF3D7_0613500	AP‐3 β	1363	0	0	0	0	0	0	0	4	2	2	96	0.78	1	2	1	79	4.74
PF3D7_1440700	AP‐3 μ3	733	0	0	0	0	0	0	0	1	2	5	88	2.48	2	3	1	92	1.93
PF3D7_1116700	DPAP1	700	5	0	0	0	2	2	44.5	19	21	20	100	0.11	18	16	13	99	0.15
PF3D7_0932300	M18AAP	570	5	3	4	0	3	4	43.2	40	43	32	99	0.08	22	23	24	98	0.4
PF3D7_1446200	LAP	605	0	0	0	10	9	100	0.01	2	4	2	96	0.93	1	2	2	89	2.22
PF3D7_0911900	falstatin	413	0	0	0	0	0	0	0	8	10	8	100	0.01	1	0	0	0	76.3
PF3D7_0934500	V_1_E	235	0	0	0	3	0	35	21.9	5	6	6	100	0.02	2	2	1	91	1.63
PF3D7_1323200	V_1_G	123	0	0	0	0	2	29	22.7	4	5	6	98	0.3	2	3	2	96	0.68
PF3D7_1311900	V_0_a	611	3	5	0	8	16	80	5.24	17	19	14	92	1.5	17	13	9	92	2.11
PF3D7_1140100	V_1_F	128	0	0	0	0	0	0	0	3	4	1	92	1.5	1	2	2	91	2.17
PF3D7_0406100	V_1_B	494	6	0	0	5	0	11	36.3	8	18	11	87	2.9	10	13	10	92	1.36
PF3D7_0914400	KIC3	249	0	0	0	0	0	0	0	2	2	2	94	1.2	0	2	0	28	17.2
PF3D7_1025000	Eps15	980	0	0	0	0	0	0	0	1	2	2	85	3.5	2	2	2	96	0.8

Abbreviations: DIX: DIX domain‐containing protein; DPAP1: Dipeptidyl aminopeptidase 1; FDR: false discovery rate; IP: Immunoprecipitation; LAP: M17 leucyl aminopeptidase; M18AAP: M18 aspartyl aminopeptidase; P: probability; R1–R3: replicate 1–3; SORTLR: Sortilin; DnaJ: DnaJ protein; V_0_a: V‐type proton ATPase catalytic subunit A; V_1_E, V_1_G, V_1_F, and V_1_B: V‐type proton ATPase subunit E, G, F, and B.

To further validate these results, we conducted reciprocal GFP‐trap pulldowns from the *Pf*CLC::GFP parasites. In three replicates, we identified 332 proteins using the same SAINT cutoffs (Table S2). Consistently, *Pf*CHC, *Pf*CLC, AP‐1, Sortilin, and Tepsin were identified at high abundance. Additionally, four AP‐4 subunits, two AP‐2 subunits (AP‐2 μ2 and AP‐2 α), and two AP‐3 subunits (AP‐3 β and AP‐3 μ3) were identified at lower abundance (Table 1, Table S2). Furthermore, some proteins related to hemoglobin digestion, including three aminopeptidases and falstatin, a cysteine protease inhibitor, were identified from the *Pf*CLC pulldowns (Table 1, Table S2). Interestingly, we also identified five subunits of the V‐type proton ATPase, which were localized at the food vacuole and parasite plasma membrane and are involved in hemoglobin uptake [37, 38] (Table 1, Table S2). Taken together, these interactome analyses revealed that clathrins were also associated with other APs (AP2‐4) and proteins involved in hemoglobin uptake and digestion.

To confirm the interaction between clathrins and AP‐1, we conducted reciprocal GFP‐trap pulldowns with the *Pf*AP‐1 γ::GFP parasite and identified 342 interacting proteins (Table S3). As expected, *Pf*CHC, *Pf*CLC, and AP‐1 were highly abundant in the pulldowns (Table 1, Table S3). Furthermore, two aminopeptidases (M18 aspartyl aminopeptidase and M1‐family alanyl aminopeptidase) and the five subunits of the V‐type proton ATPase were detected (Table 1, Table S3). Interestingly, the interactomes of *Pf*CLC and *Pf*AP‐1 γ also shared KIC3 and Eps15 with the *Pf*K13 interactome identified by BioID [18], although these proteins were relatively scarce in *Pf*CLC and *Pf*AP‐1 γ pulldowns (Table 1, Table S2, Table S3), suggesting that the *Pf*K13 complex may be loosely or indirectly associated with the clathrins‐AP‐1.

### 2.3 Clathrins Are Localized at the Parasite Periphery and the Collar of the Cytostome

**FIGURE 1:**
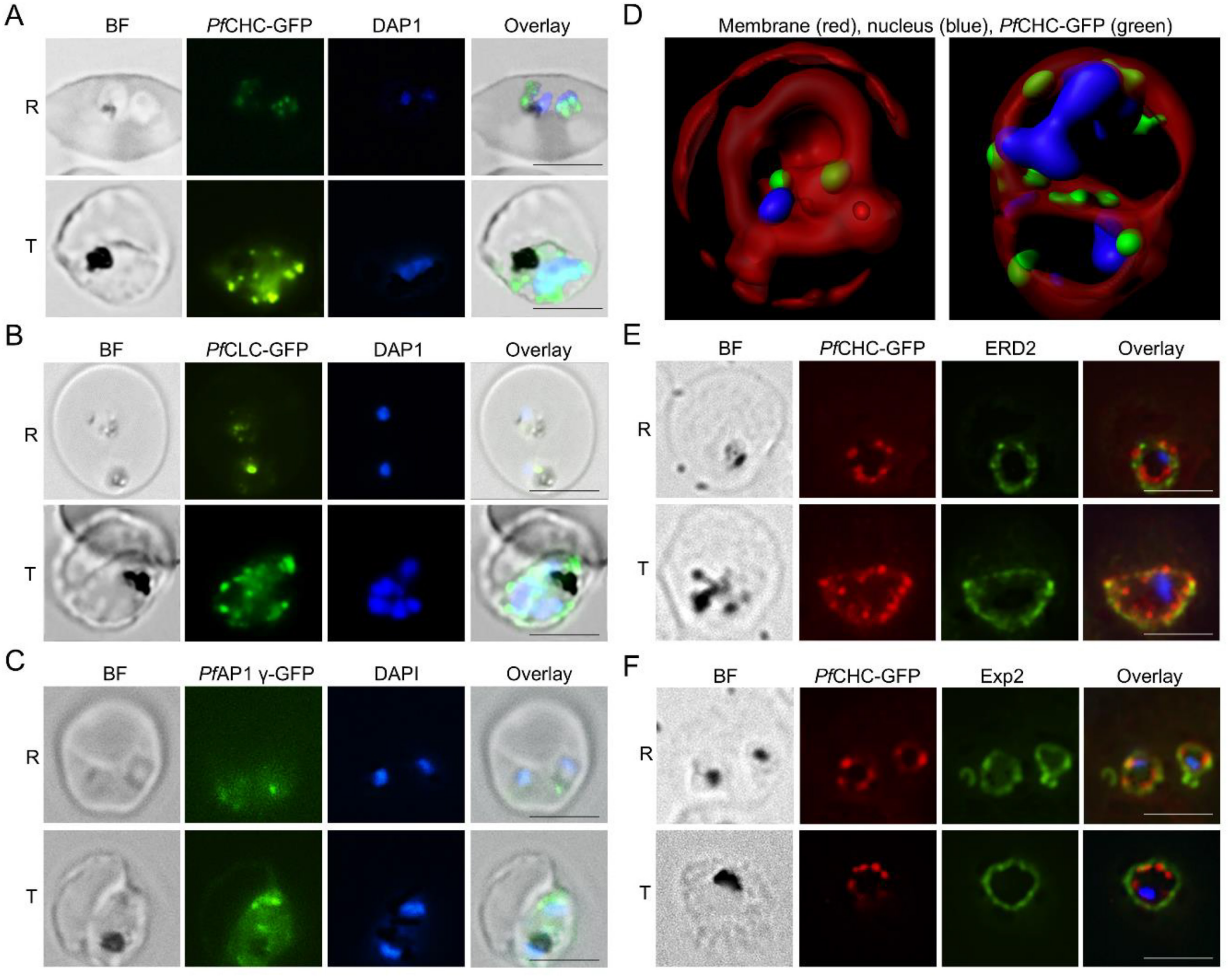
The localization of clathrins and AP‐1 γ in *P. falciparum* by IFA. (A–C) Live imaging shows GFP foci in the PfCHC::GFP (A), PfCLC::GFP (B), and PfAP‐1 γ::GFP (C) parasite lines during asexual development. BF: bright field, R: ring, T: trophozoite. The size of the scale bar is 5 μm. (D) Two representative images of PfCHC::GFP‐infected erythrocytes by structured illumination microscopy showing the three‐dimensional display of cells with Bodipy membrane staining (GFP foci in green, nucleus in blue, and membrane in red). (E and F) Representative images show the partial colocalization of PfCHC with Golgi marker ERD2 (E) and parasitophorous vacuole marker Exp2 (F) in the asexual stage parasites. The size of the scale bar is 5 μm.

To understand the potential involvement of clathrins in vesicle transport in *P. falciparum*, we first evaluated their localizations in the three GFP‐labeled parasite lines. Live‐cell fluorescence microscopy revealed the presence of large fluorescent puncta in the parasite, with the size and number of GFP puncta substantially increasing during the intraerythrocytic developmental cycle (Figure 1A–C). Under structured illumination microscopy (SIM), the GFP puncta in the parasite appeared as spheres, teardrops, or discs with variable sizes of 100–300 nm in diameter (Figure 1D, Figure S4). Intriguingly, these GFP puncta were localized at the periphery of trophozoites. Indirect immunofluorescence assay (IFA) revealed that *Pf*CHC‐GFP was juxtaposed with the cis‐Golgi marker ERD2 (Figure 1E, Pearson's correlation *R* = 0.22 ± 0.13, *n* = 23) and was within the parasitophorous vacuole marker Exp2 that defines the parasite boundary (Figure 1F).

To refine the ultrastructures of peripherally localized clathrin puncta, we used the ascorbate peroxidase 2 (APEX2)‐based transmission electron microscopy (TEM). APEX2 is an engineered ascorbate peroxidase that can convert diaminobenzidine into an insoluble polymer in the presence of H_2_O_2_ for a short time, which shows strong EM contrast at the site of the APEX2‐tagged protein after staining with osmium tetroxide (OsO_4_) [39]. The central advantage of this technology is that APEX2 remains active after glutaraldehyde fixation to offer excellent preservation of subcellular structures. The diaminobenzidine polymer remains tightly localized to the site of production and does not cross membranes, thus allowing precise localization of the labeled protein [39, 40]. Using the single crossover homologous recombination strategy, we tagged the C‐termini of the endogenous *Pf*CHC, *Pf*CLC, and *Pf*AP‐1 γ with APEX2 (Figure S5).

**FIGURE 2:**
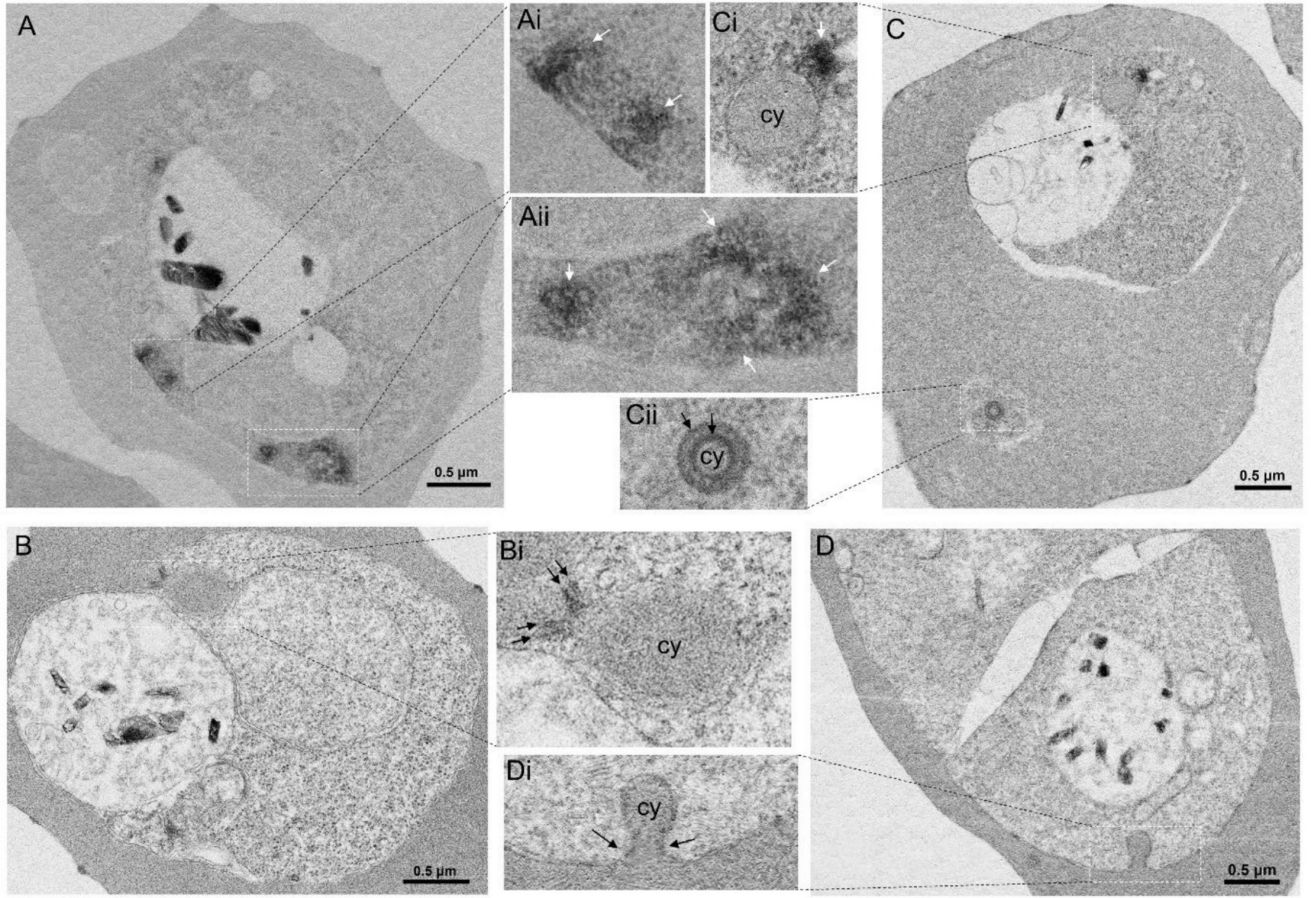
*Pf*CHC localization in *Pf*CHC::APEX2 parasites by APEX2‐based EM. (A) A representative EM image shows that electron‐dense materials coat vesicle‐like structures clustered in the parasite cytosol (white arrows in the insets Ai and Aii). (B) A representative image displays that electron‐dense materials are localized at the cytostome neck (black arrows in the inset Bi). cy: cytostome. (C) A representative EM image indicates that electron‐dense materials are localized in vesicle‐like structures in the parasite cytosol (white arrow in the inset Ci), close to the cytostome and at the collar of a horizontally sectioned cytostome (black arrows in the inset Cii). (D) A representative EM image shows electron‐dense materials were detected neither at a cytostome neck (black arrows in the inset Di) nor the parasite periphery of the non‐APEX2 tagged parasites (3D7) with the same APEX2‐based EM conditions (OsO_4_ staining after diaminobenzidine polymer reaction with H_2_O_2_).

APEX2‐based TEM analysis in the *Pf*CHC::APEX2 parasite line revealed that the electron‐dense materials were deposited in the parasite cytosol and near the parasite plasma membrane. Some electron‐dense materials formed clusters of small circles, reminiscent of the clathrin‐coated vesicles, while some electron‐dense signals did not form regular circles but accumulated closely, which could be derived from the stacked vesicles (Figure 2A, white arrows). Surprisingly, electron‐dense materials also coated the cytostome collars (Figure 2B,C, black arrows). Most of the cytostomes were vertically sectioned in the sections, showing two layers of electron‐dense material at the cytostome collars (Figure 2B). Occasionally, electron‐dense material‐labeled double rings were found in the sections (Figure 2C, black arrows), which might be the cytostome neck that was horizontally cut or other unknown structures. Furthermore, the electron‐dense coats of the vesicle‐like structures were often localized near the cytostomes (Figure 2C, white arrows). In contrast, the wild‐type parasite 3D7 under the same APEX2 reaction (diaminobenzidine and H_2_O_2_) and OsO_4_ staining did not show any electron‐dense materials at the parasite periphery and cytostome collar (Figure 2D, black arrows). The same patterns of electron‐dense materials were also identified in the *Pf*CLC::APEX2 parasite line (Figure S6).

**FIGURE 3:**
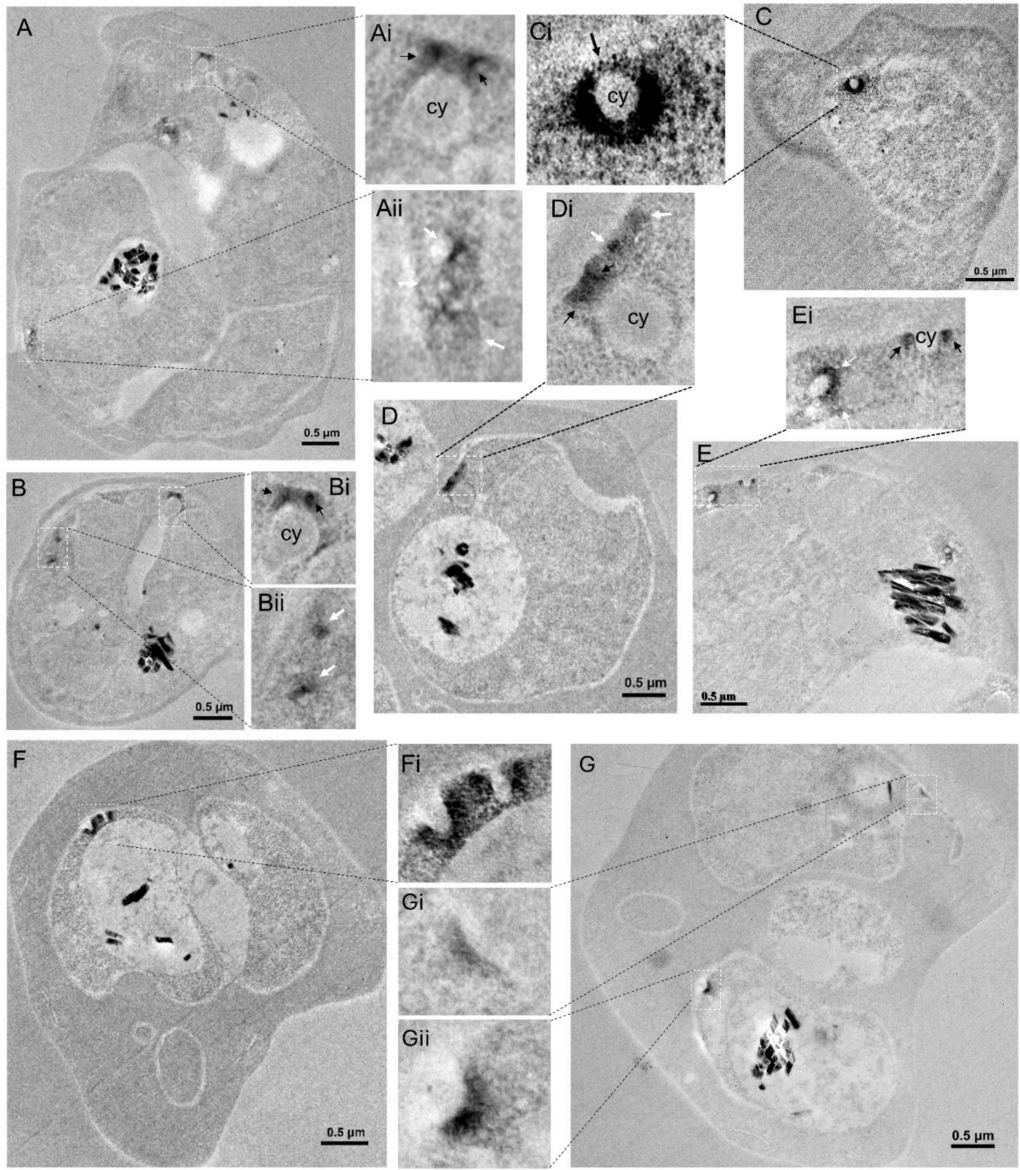
*Pf*AP‐1 γ localization in *Pf*AP‐1 γ::APEX2 parasites by APEX2‐based EM. (A and B) Two representative EM images show that electron‐dense materials at the cytostome neck (black arrows in the inserts Ai and Bi) and vesicle‐like structures clustered in the parasite cytosol (white arrows in the inserts Aii and Bii) cy: cytostome. (C) A representative image indicates a horizontally sectioned cytostome with a single layer of electron‐dense materials at its collar (black arrow in the insert Ci). (D and E) Two representative EM images display that electron‐dense materials are localized in vesicle‐like structures in the parasite cytosol close to cytostomes (white arrows in the inserts Di and Ei) and at the collar of the cytostome (black arrows in the inserts Di and Ei). (F and G) Two EM images show that the electron‐dense materials coat the outer surface of two parallel small invaginations (early‐stage cytostomes) (F and its inset Fi) and two separated small invaginations (G and its insets Gi and Gii) from the parasite plasma membrane.

We then performed APEX2‐based TEM analysis in the *Pf*AP‐1 γ::APEX2 parasite line. Like the localization of *Pf*CHC and *Pf*CLC, *Pf*AP‐1 γ was also localized at vesicle‐like structures near the parasite plasma membrane (Figures 3A, 2B, Figure S7A–S7C, white arrows). In contrast to the double layers of *Pf*CHC and *Pf*CLC coats at the cytostome necks, only a single layer of electron‐dense materials was detected at the cytostome collars in the *Pf*AP‐1 γ::APEX2 parasite line when the cytostomes were sectioned vertically (Figure 3A,B,D,E, Figure S7E, black arrows) or horizontally (Figure 3C, Figures S7C, S7D, black arrows). Likewise, the electron‐dense materials‐coated vesicles were often localized near the cytostomes (Figure 3D,E, Figure S7E, white arrows). Additionally, the electron‐dense materials were found to coat the entire surface of small invagination at the parasite plasma membrane (Figure 3F,G). Two parallel, electron‐dense material‐coated invaginations in Figure 3F were reminiscent of the clusters of cytostomes observed by ultrastructural expansion microscopy [25], indicating that these are likely early‐stage cytostomes.

### 2.4 CCVs In Malaria Parasites Appear More Diverse

**FIGURE 4:**
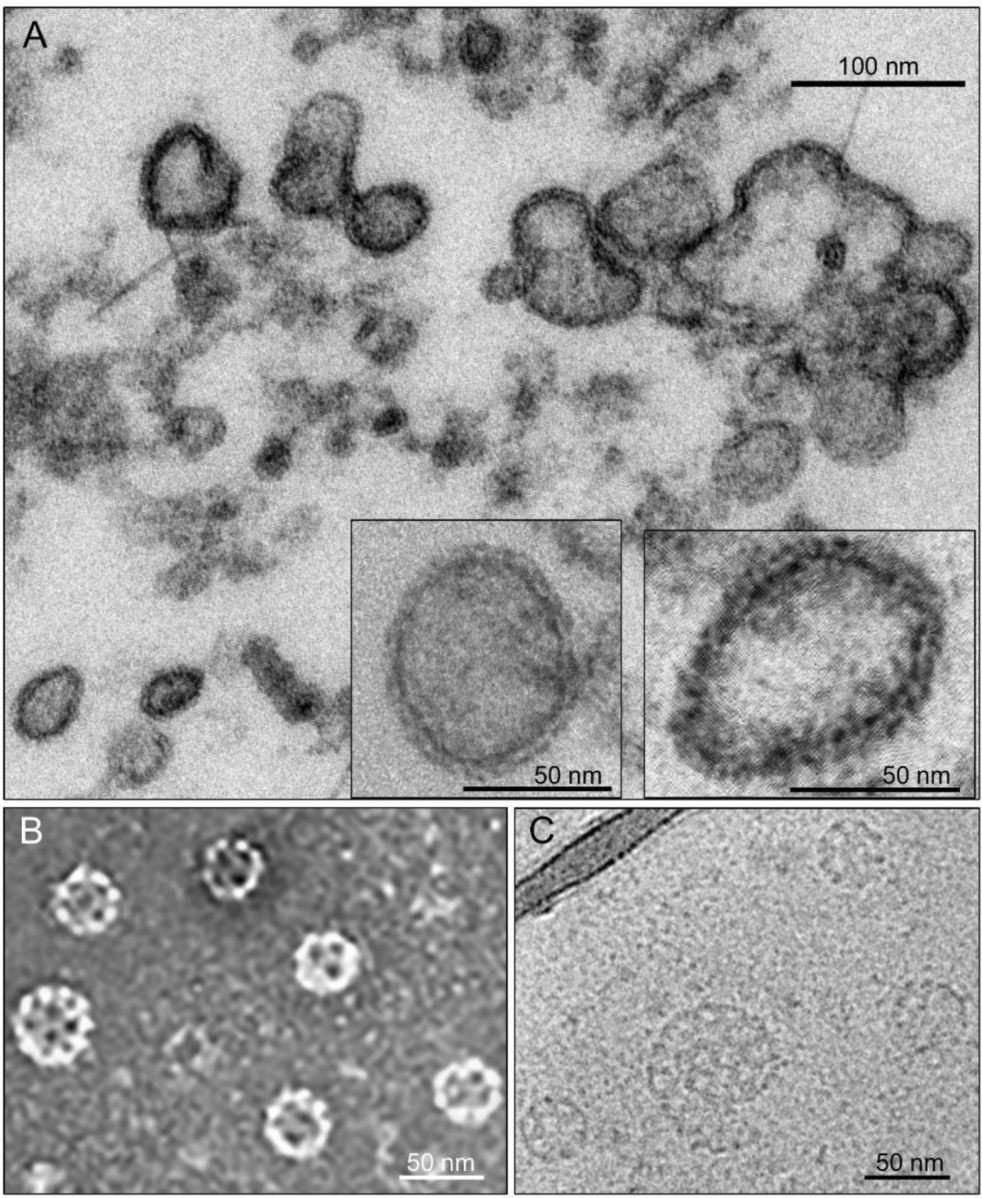
Morphology of CCVs in *P. falciparum*. (A) Transmission EM image of CCVs purified by GFP‐trap beads from PfCHC::GFP parasites. Two inserts show two smaller CCVs. (B and C) Transmission EM (B) and Cryo‐EM (C) images of CCVs purified by ultracentrifugation from wild‐type 3D7 parasite after negative staining (B) and cryofixation (C).

APEX2‐based TEM easily identified electron‐dense material‐coated CCV‐like structures in the parasite (Figures 2 and 3, Figures S6 and S7). This is in stark contrast to the rare report of CCVs in malaria parasites [41]. We used two approaches to further elucidate the structure of CCVs in *P. falciparum*. First, we purified CCVs from the *Pf*CHC::GFP parasite using anti‐GFP magnetic beads, followed by negative staining TEM analysis. We detected vesicles with conspicuous coat structures, many similar in size to canonical CCVs, while others were much larger and pleomorphic (Figure 4A, Figure S8A). The thickness of the electron‐dense coat is ~7 nm, significantly thinner than that in CCVs of mammalian cells (~15 nm) [7, 42]. This coat could be mistaken for double membrane‐bound vesicles (Figure S8B). Secondly, we harvested CCVs from wild‐type parasites by sucrose/Ficoll‐gradient ultracentrifugation, a technique commonly used to prepare homogeneous CCVs from mammalian cells [43]. TEM and cryoEM revealed typical CCV‐like vesicles with an average size of 36.9 nm (30–50 nm, *n* = 104) in the harvested materials (Figure 4B,C). Analysis of the harvested materials by liquid chromatography and tandem mass spectrometry detected many proteins, including *Pf*CHC, *Pf*CLC, and *Pf*AP‐1 subunits at high abundance (Table S4), consistent with *Pf*CHC and *Pf*CLC affinity purifications (Table 1). Many other proteins were also identified from purified vesicles, including proteins involved in vesicle transferring, cargo trafficking in CCVs and other vesicles (COPI and COPII), since sucrose/Ficoll‐gradient‐based purification normally will harvest any small‐sized vesicles.

### 2.5 *Pf*CHC KD Leads to Reduced Hemoglobin Uptake

**FIGURE 5:**
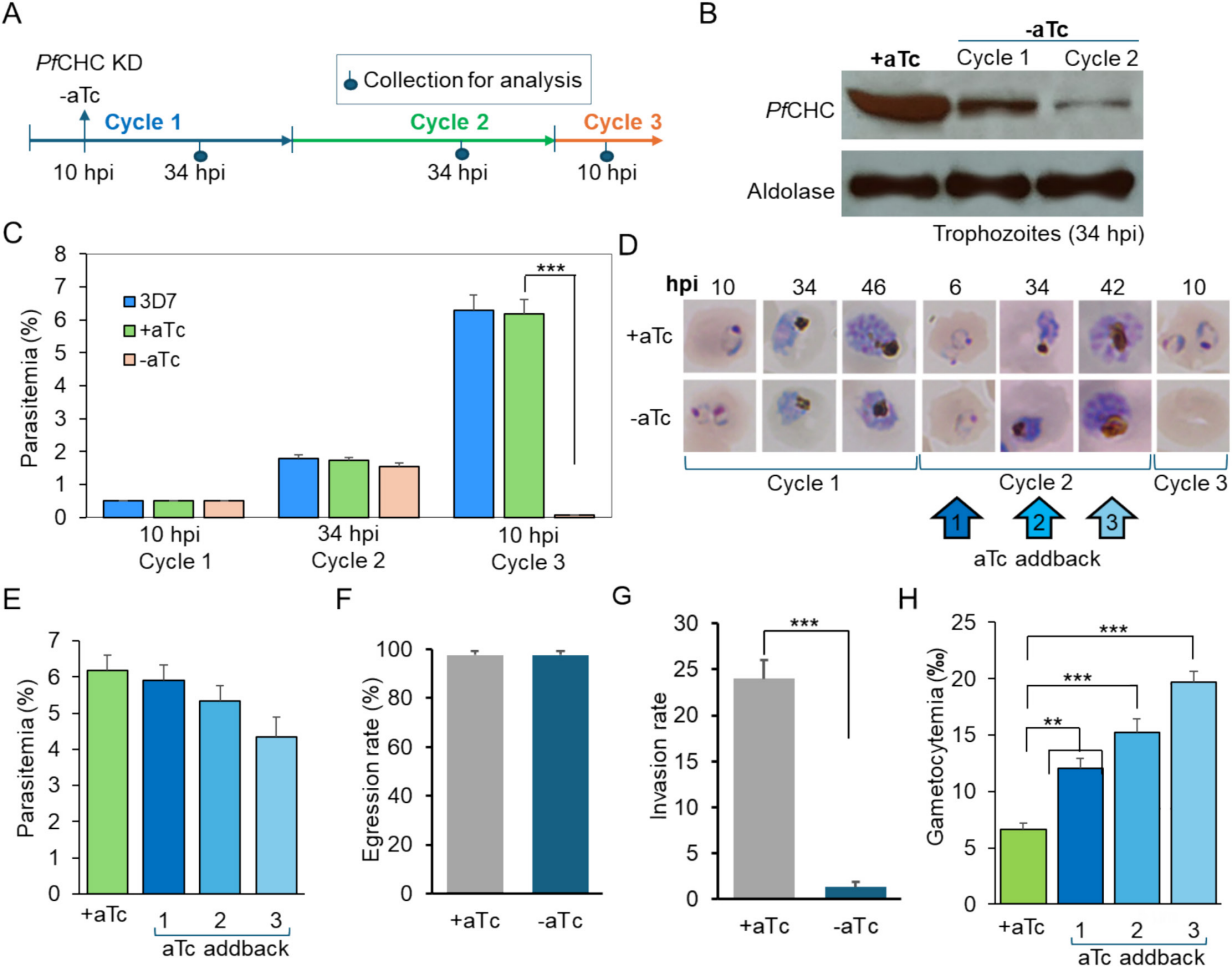
Growth phenotypes and protein export upon *Pf*CHC KD. (A) A schematic diagram shows the experimental workflow on the analysis of PfCHC expression (B) and growth (C–F) after PfCHC KD. (B) Western blot with anti‐GFP antibodies showed the progressive reduction of PfCHC‐GFP protein level in cycle 1 (~60%) and cycle 2 (~90%). aTc was withdrawn from the culture at 10 h post‐invasion (hpi). The intensities of protein bands were measured using ImageJ. (C and D). Parasite growth after PfCHC KD by measuring parasitemia (C) and checking parasite development by Giemsa staining (D). The starting parasite cultures at 0.5% ring grew normally in cycle 2, but no ring‐stage parasites appeared at 10 hpi in cycle 3. ***p < 0.001. (E) When aTc was added back to the culture in cycle 2 at the ring, trophozoite, or schizont stage (6, 34, or 42 hpi), a variable percentage of parasites could survive in the next cycle. F and G. Egression rate (F) was not changed, but invasion rate (G) was substantially reduced in merozoites at the second cycle upon PfCHC KD (−aTc) compared to the wildtype parasites (+aTc). (H) The higher levels of gametocytemia appeared after PfCHC KD (−aTc) compared to wildtype (+aTc) (three replicates, **, ***: *p* < 0.01, 0.001, respectively, *t*‐test). aTc was withdrawn from the culture at 10 h post‐invasion (hpi) in the first cycle and added back at three time points in the second cycle, as shown in D. Gametocytemia was measured 5 days after the culture was incubated with heparin for 3 days, starting at the time of adding back aTc to the culture to remove any asexual stage parasites.

To understand clathrin function, we attempted to disrupt *PfCHC* by single and double crossover recombination but without success, consistent with the essentiality of this gene from transposon mutagenesis analysis [44]. We then applied the TetR‐DOZI conditional KD system to reduce the expression of *Pf*CHC‐GFP [45, 46] (Figures S9A, S9B). In the engineered parasite TetR‐*Pf*CHC::GFP, withdrawing anhydrotetracycline (aTc) from the 10‐h post invasion (hpi) ring‐stage parasites for about 24 h (defined as 34 hpi trophozoites in Cycle 1), *Pf*CHC expression was reduced to ~40% (Figure 5A,B). Culturing the TetR‐*Pf*CHC::GFP parasites without aTc for an additional 48 h to the 34 hpi trophozoite stage of Cycle 2 resulted in approximately 90% reduction of *Pf*CHC expression (Figure 5A,B). Compared to the TetR‐*Pf*CHC::GFP parasite cultured in parallel with constant aTc, parasite growth was only slightly reduced after aTc withdrawal in Cycle 1, and the majority of parasites developed into mature schizonts. However, in the ring stage (10 hpi) of Cycle 3 without aTc, when all schizonts had egressed, we observed a very low number of ring‐stage parasites in the culture. In contrast, parasites cultured in parallel with aTc all developed into the ring stage (Figure 5C,D). These results suggested that merozoites egressed from the schizonts of Cycle 2 were defective in the invasion of RBCs. Adding aTc back into the culture at the ring, trophozoite, and schizont stage in Cycle 2 enabled parasite growth into Cycle 3 at ~95%, ~86%, and ~70% efficiency compared with parasites cultured with constant aTc, confirming that the observed invasion defect was due to *Pf*CHC KD (Figure 5D,E). Merozoite egression and invasion rates were measured using schizonts from the second cycle. As we expected, the egression rate was not altered, whereas the invasion rate was significantly reduced after *Pf*CHC KD (Figure 5F,G). Intriguingly, the later aTc was added back to the culture, the higher levels of gametocytes appeared in the culture (Figure 5H), indicating that *Pf*CHC KD led to increased commitment to gametocytogenesis, probably due to the stress conditions induced by *Pf*CHC KD.

The location of clathrin‐coated vesicle‐like structures at the parasite periphery (Figures 2 and 3, Figures S6 and S7) suggests that clathrins may be involved in protein export into RBCs. To investigate whether *Pf*CHC KD influences this process, we performed IFA on two PEXEL motif‐containing proteins (KAHRP and FIKK4.2) and four PEXEL‐negative exported proteins (PfEMP1, REX1, REX3, and SBP1) [47, 48] after aTc withdrawal at the ring stage in the first cycle for 48–60 h. No discernible reduction in the export of these proteins was detected (Figure S9C).

**FIGURE 6:**
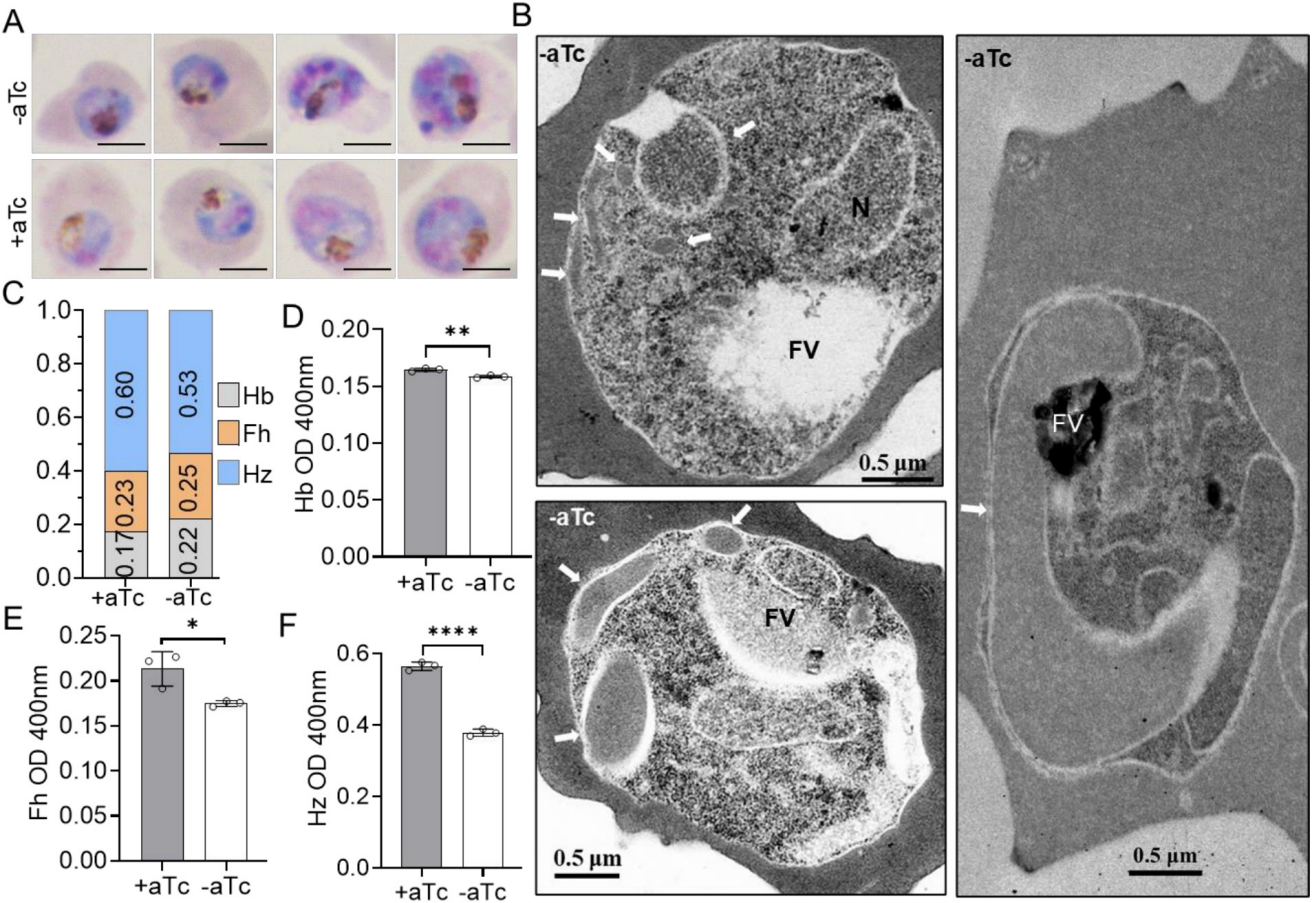
*Pf*CHC KD led to abnormal cytostome and lower Hb uptake and digestion. (A) Representative Giemsa staining images show the hemozoin blocks before (+aTc) and after (−aTc) PfCHC KD. The size bar: 5 μm. (B) Three representative EM images show the abnormal cytostome (long tubes) (white arrows) after PfCHC KD (−aTc). FV: food vacuole. (C) A graph shows the percentages of hemozoin (Hz), hemoglobin (Hb), and free heme (Fh) in the synchronized parasites at the trophozoite stage before (+aTc) and after (−aTc) PfCHC KD. (D–F) Bar graphs display the levels of hemoglobin (D), free heme (E), and hemozoin (F) in the trophozoites before (+aTc) and after (−aTc) PfCHC KD. *, **, ****: *p* < 0.05, 0.01, and 0.0001, respectively, *t*‐test.

The localization of clathrins at cytostome collars suggests their potential involvement in endocytosis. In Giemsa‐stained smears, we observed that the hemozoin pigment in the food vacuole (FV) of the *Pf*CHC KD parasite 72 h post‐aTc withdrawal from the ring in the first cycle to trophozoite in the second cycle was much lighter in color and occupied a smaller area than in the control (Figure 6A, Figure S9D), suggesting the accumulation of a lower amount of hemozoin in *Pf*CHC KD parasites. TEM revealed long tube‐like cytostomes in the *Pf*CHC KD parasite (−aTc) compared to the hemoglobin‐containing small vesicles in the control parasite (+aTc), indicating that *Pf*CHC KD led to a defect in the pinching off of hemoglobin‐containing vesicles from cytostomes and therefore caused less amount of hemoglobin to be delivered to the food vacuole (Figure 6B, Figure S9E).

From equal numbers of synchronized *Pf*CHC‐KD and control parasites at the trophozoite stage, we quantified heme from hemoglobin in parasite cytosol, free heme, and hemozoin in FV by cell fractionation and measuring heme–pyridine complex [49, 50]. The results showed that hemozoin is the major source of heme (~60%) while ~20% heme was derived from hemoglobin and free heme, respectively (Figure 6C). *Pf*CHC‐KD parasites (−aTc) contained a significantly lower amount of hemoglobin (Figure 6D), free heme (Figure 6E), and hemozoin (Figure 6F) than control parasites (+aTc). Consistently, another hemozoin measurement by only NaOH dissolution resulted in the same trend [51, 52] (Figure S9F).

**FIGURE 7:**
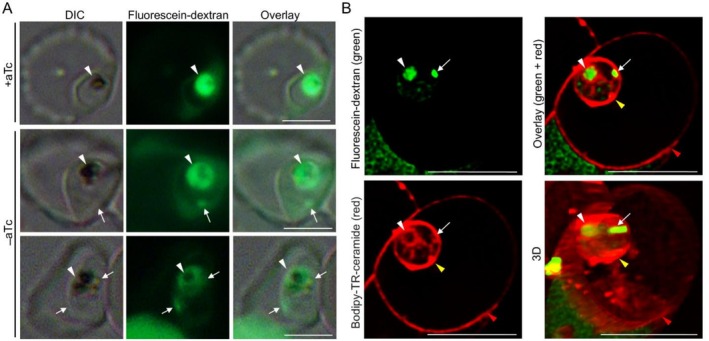
Abnormal Hb containing structures upon *Pf*CHC KD. (A) Representative images show the dextran (green)‐containing structures in the parasites under fluorescence microscopy. The white arrows indicate the dextran‐containing structures in the PfCHC KD parasites (−aTc), whereas no such structure was seen in the wildtype parasites (+aTc). The white arrowheads denote the hemozoin segments, which overlap with strong dextran signals where the food vacuole is located in each parasite. DIC: Differential Interference Contrast. The size bar: 5 μm. B. Representative super‐resolution images display the dextran‐containing structures in a PfCHC KD parasite (−aTc) with membrane staining by lipid dye (Bodipy‐TR‐C5_ceramide). The signals from dextran (green), membrane (red), and their overlay in the parasite‐infected red blood cell are shown from a single confocal z stack (section), respectively. A three‐dimensional (3D) reconstructed image, which was built from all z stacks, is shown from one angle. Please see Video S1 for 3D images from all angles. The white arrow and arrowhead indicate two dextran (green)‐containing membrane (red) structures, one *showing* a food vacuole‐like globally structure while another displays a long‐tubular‐like structure (abnormal cytostome). A yellow arrowhead indicates the parasite membrane, while a red arrowhead denotes the red blood cell membrane. The size bar: 5 μm.

Since the mouth of the cytostome is open to the RBC cytosol, lysis of the RBC membrane will release hemoglobin from both RBC and cytostomes but not from the hemoglobin‐containing vesicles that have pinched off from cytostomes. Thus, if the lysis of the RBC membrane releases hemoglobin from abnormally long tube‐like cytostomes in the *Pf*CHC KD parasites, the hemoglobin content in the *Pf*CHC KD parasites will be lower than in the control. To verify this, resealed RBCs containing fluorescein‐dextran were infected by *Pf*CHC KD parasites. Flow cytometry indicated that fluorescence signals in *Pf*CHC KD parasites (‐aTc) were significantly lower than their controls (+aTc) (Figure S9G, *p* < 0.05, *t*‐test). Furthermore, under fluorescence microscopy, *Pf*CHC KD parasites displayed additional dextran signals besides the strong dextran signals in the food vacuole, which were highly overlapped with hemozoin segments, whereas wildtype parasites only showed food vacuole‐localized fluorescence signals (Figure 7A, Figure S10A), in agreement with the abnormal, elongated cytostomes revealed by TEM (Figure 6B, Figure S9E). Furthermore, confocal microscopy with LIGHTNING deconvolution‐based super‐resolution mode after using lipid dye (Bodipy‐TR‐C5‐ceramide) further confirmed this finding with three‐dimensional reconstruction showing long, tube‐like membrane structures containing dextran in the *Pf*CHC KD parasite (Figure 7B, Figure S10B, Videos S1 and S2).

## 3 Discussion

In this study, we studied the interactome, localizations, and functions of clathrins in *P. falciparum*. Interactome studies confirmed the association of clathrins mainly with the AP‐1 complex and, to a much lower extent, with other AP complexes. In addition, we identified their associations with proteins involved in hemoglobin uptake/digestion. IFA and APEX2‐based TEM precisely identified the localization of *Pf*CHC, *Pf*CLC, and *Pf*AP‐1 γ at vesicle‐like structures, which were clustered together near the parasite plasma membrane and at the collar of the cytostome. *Pf*CHC KD led to defects in merozoite invasion, the accumulation of abnormal cytostome structures, and consequently reduced hemoglobin digestion.

Same as *Pf*CHC‐BioID pulldown [18], we also did not detect AP2 and KIC3 in GFP‐trap pulldown from *Pf*CHC::GFP parasite line. However, we identified these proteins in GFP‐trap pulldown from *Pf*CLC::GFP parasite line. One significant difference between our pulldowns from *Pf*CHC::GFP and *Pf*CLC::GFP is the abundances of *Pf*CHC and *Pf*CLC from *Pf*CLC::GFP parasites were ~3.5 and 4.4‐fold higher than those from *Pf*CHC::GFP parasites, respectively (Table 1), indicating that *Pf*CLC‐GFP is easier to access by GFP‐trap beads than *Pf*CHC‐GFP, probably because *Pf*CLC‐GFP is at the surface of CCV, whereas GFP‐tagged *Pf*CHC carboxy‐terminal end is underneath the surface of CCV because a clathrin triskelion is linked by three carboxy‐terminal segments of *Pf*CHC, which bend inward to form a threefold axis or vertex [2]. Similarly, clathrins were not identified in the pulldown by anti‐GFP magnetic beads from AP2 μ::GFP parasites [17], probably because AP2 μ‐GFP is also underneath the surface of CCV [3]. Additionally, GFP‐trap beads, using anti‐GFP nanobody without heavy and light chains, allow a high capacity of pulldown with low background. Thus, GFP‐trap pulldown enhanced the chances of identifying AP2 and KIC3 from *Pf*CLC::GFP parasites. Furthermore, a recent study on Tepsin interactome revealed that Tepsin interacts with *Pf*CHC, *Pf*CLC, and AP‐4 ε [53], which is consistent with our *Pf*CLC interactome data.

It is conceivable that *Pf*AP‐1‐based CCVs near the Golgi mediate secretion and trafficking between the Golgi and endosomes in the parasite. Since protein export to the host RBC cytosol was not noticeably altered upon *Pf*CHC KD, *Pf*AP‐1‐containing CCVs may be primarily involved in protein secretion to the parasite plasma membrane or parasitophorous vacuole for cytostome biogenesis and protein trafficking between the Golgi and endosomes (cytostome and food vacuole). Since Sortilin was also identified in the clathrin IPs from the parasite, clathrin/AP‐1‐based transport may have a conserved function in protein transport among the Golgi, endosome, and parasite plasma membrane (exocytosis) [54, 55, 56, 57]. Previous reports showed that some hemoglobin proteases, such as falcipain and plasmepsin II, were found to be transported to the parasitophorous vacuole or cytostome *en route* to the food vacuole [58, 59, 60, 61]. Two recent studies also showed that the V‐type ATPase, which regulates the pH of the food vacuole by pumping protons and consists of eight cytosolic V_1_ domain‐containing subunits and five membrane‐embedded V_0_ domain‐containing subunits, was localized at the food vacuole and parasite plasma membrane [37, 38]. KD of its subunits resulted in accumulated hemoglobin‐containing vesicles in the food vacuole, indicating that this complex is also potentially transported to the cytostome to create acidic conditions en route to the food vacuole [37, 38]. Interestingly, previous pulldowns using anti‐GFP magnetic beads [17] and our current GFP‐trap pulldown studies identified several proteases, a protease inhibitor (falstatin), and subunits of the V‐type ATPase, suggesting that they may be cargoes of AP‐1‐based CCVs. It is of great interest to identify the cargoes of CCV‐AP1 in *P. falciparum*.

Conventional AP‐2‐based CCVs in eukaryotes mediate endocytosis. However, recent studies indicated that AP‐2 appeared not to mediate endocytosis via the cytostome in malaria parasites [17, 18]. It is difficult to understand how the AP‐2 complex works without clathrins in mediating endocytosis since, so far, endocytosis requiring AP‐2 but not clathrin has been reported only in *Aspergillus* [20, 23, 62]. A significant finding in this study is that clathrins are also involved in hemoglobin uptake. First, *Pf*CHC, *Pf*CLC, and *Pf*AP‐1 γ were localized at the collar of the cytostome. Notably, studies in *T. gondii* have provided evidence supporting the localizations of clathrins and AP‐1 at the micropore [63, 64, 65]. A TEM report revealed a clathrin‐like coat at the micropore surface [63]. Two IFA studies revealed that both *Tg*CHC and *Tg*AP‐1 μ1 were localized at the parasite plasma membrane, and *Tg*CHC was in the openings of the inner membrane complex, the locations of micropores [64, 65] (Figures S11A, S11B). Second, *Pf*CHC KD resulted in abnormal, long cytostome tubes, indicating *Pf*CHC may be directly or indirectly involved in the pinch‐off of the cytostome, leading to reduced hemoglobin digestion. Third, affinity pulldowns of clathrins identified lower amounts of the *Pf*K13 complex (KIC3 or Eps15) and AP‐2 components, suggesting that the association may be loose or highly dynamic. Last, when we revisited the previous co‐localization study by IFAs [18], we found that *Pf*CHC was closely juxtaposed with *Pf*K13 at the parasite boundary (Figure S11C). Recently, reports showed that *Pf*K13 was also found at the cytostome collar and involved in the cytostome process [24], and *Pf*K13 was colocalized with AP‐2 μ [17, 18]. The exact relationships among *Pf*K13, clathrins, AP‐2, and AP‐1 in the cytostome process are unknown and worthy of investigation in the future. Currently, we do not have any clue about the reason why *Pf*CHC forms a double layer while *Pf*CLC forms a single layer at cytostome necks. The single layer of *Pf*CLC seems to be localized at the same location as the inner layer of *Pf*CHC. We assume that the inner layer is important for the cytostome to be constrained and pinched off, while the outer layer may be the assembling site for the inner layer. When *Pf*CLC is assembled with *Pf*CHC at the outer layer, the assembled CHC/CLC will be immediately transferred to the inner layer.

After cytostomes pinch off, hemoglobin‐containing vesicles, equivalent to early endosomes, either mature to become a food vacuole in the parasite at the early asexual developmental stage or fuse with an existing food vacuole at the late stage [61, 66, 67]. Several factors, including actin‐myosin, dynamin, SNARE, Rab5a, and VPS45, were found to be involved in these processes [61, 67, 68, 69]. Inhibition of actin destabilization and the activities of dynamin, myosin, and SNARE resulted in hemoglobin‐filled long cytostome tubes, whereas inhibition of actin stabilization and mislocalization of VPS45 caused an accumulation of hemoglobin‐containing vesicles in the parasite, indicating that these factors are needed for the pinch‐off of hemoglobin‐containing vesicles and their subsequent transport to the food vacuole, respectively [61, 67, 68, 69]. The similar hemoglobin‐filled long cytostome tubes after *Pf*CHC KD suggest that clathrins are also involved in the cytostome pinch‐off in *P. falciparum*.

Our EM analysis showed CCV‐like polygonal cells in the CCVs purified from parasites. However, the thickness of the electron‐dense coat of CCVs of the parasite is significantly thinner than CCVs in mammalian cells [7, 42], probably due to the highly diverse domain structures of clathrin and AP subunits in the parasite. However, these observations could be artefactual and need to be further verified because we did not directly compare the thickness of mammalian cells' CCVs by using the same TEM staining in this study. *Pf*CHC KD caused a significant defect in merozoite invasion, indicating that clathrin is critical for this process. Previous studies showed that *Pf*AP‐1 μ1 was colocalized with rhoptry [16], and clathrin‐based transport was found to be vital for exporting proteins from the Golgi to the invasion‐related apical secretory organelle (rhoptry and microneme) in *T. gondii* [14, 64, 65]. Thus, it is of great interest to elucidate the functions of clathrins in this secretory pathway.

Although this study showed that clathrins and AP1 localize to cytostome necks and that *Pf*CHC knockdown leads to abnormal cytostome formation and impaired hemoglobin uptake, suggesting that clathrins are involved in this critical endocytosis process, this study did not provide direct evidence of whether or how clathrin participates in cytostome function. Further in‐depth analysis will be needed to validate and clarify these findings.

## 4 Materials and Methods

### 4.1 Parasite Culture

*P. falciparum* clone 3D7 was cultured in type O^+^ red blood cells as described [70]. Parasites were synchronized by treating the ring‐stage parasites with 5% D‐sorbitol.

### 4.2 Phylogenetic Comparison

GenBank entries of CHC, CLC, and AP‐1 subunits in model eukaryotes were retrieved for phylogenetic analysis. Sequence alignment and phylogenetic analysis were performed using the CLUSTAL Omega program [71].

### 4.3 Genetic Manipulation of *Pf*CHC, *Pf*CLC, and *Pf*AP‐1‐γ

To tag the C‐terminus of *Pf*CHC with GFP, a *PfCHC* fragment was amplified using primer pair 1 from the *P. falciparum* genomic DNA and cloned into a modified pBluescript SK plasmid to fuse with the GFP and pDT 3′ UTR [72, 73] (Table S5). This cassette was then subcloned into pHD22Y at the *Bam*HI and *Not*I sites to produce pHD22Y/*Pf*CHC‐GFP [74]. For APEX2 tagging, the *APEX2* fragment was amplified using primer pair 2 (Table S5). The GFP fragment in the pHD22Y/*Pf*CHC‐GFP plasmid was replaced by the APEX2 tag to generate the pHD22Y/*Pf*CHC‐APEX2. The same methods were used to tag the C‐terminus of *Pf*CLC and *Pf*AP‐1 γ with GFP or APEX2 tag using primer pair 3 and 4, respectively (Table S5), to obtain pHD22Y/*Pf*CLC‐GFP, pHD22Y/*Pf*CLC‐APEX2, pHD22Y/*Pf*AP‐1 γ‐GFP, and pHD22Y/PfAP‐1 γ‐APEX2. To knock down *PfCHC* using the TetR‐DOZI system, the *Pf*CHC C‐terminal fragment fused with GFP was amplified from pHD22Y/*Pf*CHC‐GFP by using primer pair 5 and cloned into pMG75 upstream of the 10× aptamer at *BssH*II and *Dra*III sites to obtain the final construct pMG75/*Pf*CHC‐GFP (Table S5). Parasite transfection was performed using an RBC loading method [75]. The integration was screened by PCR using a forward primer upstream of the homologous recombination region and a reverse primer located in the GFP or APEX2 tag (Table S5).

### 4.4 IPs and Mass Spectrometry

For GFP‐tagged proteins, IPs were performed using the GFP‐Trap agarose according to the manufacturer's protocol (ChromoTek). The 3D7 parasites were used as IP controls. The elutes were separated briefly in an SDS‐PAGE gel, and proteins in the gel were excised and digested as described [76]. The digests were analyzed by LC/MS/MS by the established methods using a Waters NanoAcquity HPLC system interfaced with a Q Exactive Hybrid Quadrupole‐Orbitrap Mass Spectrometer (Thermo Scientific) [77, 78, 79, 80].

### 4.5 Immunoblotting

To analyze the expression of *Pf*CHC, *Pf*CLC, and *Pf*AP‐1 γ in *Pf*CHC::GFP, *Pf*CLC::GFP, *Pf*AP‐1 γ::GFP, *Pf*CHC::APEX2, *Pf*CLC::APEX2, and TetR‐CHC::GFP parasite lines, protein extracts from the respective parasite lines at different stages were analyzed by SDS‐PAGE and immunoblotting using anti‐GFP mAb (1:1000, Roche, 11 814 460 001), anti‐APEX2 antibodies (1:1000, Innovagen, PA‐APEX2‐100), respectively, as primary antibodies and horseradish peroxidase‐conjugated IgG (1:3000) as the secondary antibody.

### 4.6 Immunofluorescence Assay (IFA)

Thin smears of iRBCs were prepared on glass slides and fixed with acetone for 10 s. Then, the slides were incubated with various antibodies, including ERD2 (rabbit, 1:500), PfEMP1 (rabbit anti‐ATS, 1 μg/mL), REX1 (rabbit, 1:5000), REX3 (rabbit, 1:1000), SBP1 (mouse, 1:1000), KAHRP (mouse mAb 18.2, 1:500), and FIKK4.2 (mouse, 1:500) [81] as primary antibodies and then incubated with FITC conjugated anti‐rabbit or mouse IgG (1:1000) (Sigma, AP132F, AP124F) antibodies as secondary antibodies. The anti‐PfEMP1 antiserum was raised against the peptide DITSSESEYEELDINDIC in PfEMP1 conserved acidic terminal (ATS) (Proteintech Group), which has been proven to detect all PfEMP1 [82]. For the colocalization study, the *Pf*CHC::GFP iRBCs were used with anti‐GFP (mouse mAb 1:1000, Roche, 11 814 460 001, rabbit ab6556, 1:1000), ERD2 (rabbit, 1:500, MR4), and Exp2 (mouse mAb 7.7, 1:500) [83]. The slides were observed under a Nikon Eclipse E600 microscope.

### 4.7 Super‐Resolution Microscopy

For structured illumination microscopy (SIM), *Pf*CHC::GFP iRBCs were stained with Bodipy‐TR‐C5‐ceramide, mounted in the Antifade Mountant with DAPI (Molecular Probes, P36970), and visualized using a Nikon N‐SIM/N‐STORM microscope. Three‐dimensional (3D) reconstructions and isosurface models in volume images of reconstructed stacks were performed using the IMARIS version 7.7.2 software suite (Bitplane AG). For the clarity of display, deconvolved z stacks were reconstructed in 3D with interpolation. For confocal super‐resolution fluorescence microscopy using deconvolution, parasite‐infected RBCs, which were resealed after fluorescein‐dextran incorporation (see below), were stained with Bodipy‐TR‐C5‐ceramide and DAPI and imaged on a Leica Stellaris 8 confocal microscope equipped with LIGHTNING deconvolution‐based super‐resolution mode. Images were acquired using a 63×/1.40 NA oil immersion objective with HyD detectors, at a voxel size of 0.02 × 0.02 × 0.27 μm (XYZ), and z‐step intervals of 0.3 μm. The LIGHTNING algorithm was applied with automated settings optimized for high signal‐to‐noise ratio (SNR), using adaptive point spread function (PSF) estimation. 3D reconstructions were performed in LAS X 3D Viewer with interpolation.

### 4.8 Conventional EM, APEX‐Based EM

Conventional TEM was performed as described [72]. The APEX2‐based TEM was conducted as described [39]. The diaminobenzidine polymer reaction was induced by applying 0.5 mM H_2_O_2_ to the iRBC samples for 1 min at 4°C, followed by 2% OsO_4_ staining for 30 min on ice with or without uranyl acetate staining. Samples were imaged using a JEOL electron microscopy.

### 4.9 Purification of CCVs

CCVs were purified using GFP‐Trap magnetic beads (Chromoteck) from *Pf*CHC::GFP parasites or by ultracentrifugation coupled with sucrose/Ficoll gradient from 3D7 parasites [43, 84]. Briefly, trophozoites were homogenized in MES buffer [100 mM MES, 1 mM EGTA, and 0.5 mM MgCl_2_ (pH 6.7)] and then centrifuged at 17 000 × *g* for 25 min, and the supernatant was transferred to either incubate with GFP‐Trap magnetic beads for 2 h or further centrifuged at 56 000 × *g* for 1 h. The GFP beads were washed with MES buffer and were ready for TEM analysis. The pellet from the above centrifugation (56 000 × *g* for 1 h) was suspended in MES buffer with 12.5% (w/v) of sucrose and Ficoll, then spun at 43 000 × *g* for 1 h. The supernatant was transferred to new tubes and centrifuged at 100 000 × *g* for 2.5 h. A small part of the pellets was resuspended in MES buffer for observation by TEM after negative staining or cryofixation. The rest of the pellets (90%) were resuspended in lysis buffer (10 mM Tris‐Cl pH 7.5, 150 mM NaCl, 0.5 mM EDTA, 0.5% Nonidet P40 Substitute) for proteomic analysis.

### 4.10 Negative Staining and cryoEM Analysis of Purified CCVs

Negative staining was performed according to a standard protocol with uranyl acetate [85]. Briefly, 3 μL of the sample was applied onto a glow‐discharged carbon‐coated grid (Ted Pella) and incubated for 1 min. The sample was blotted with filter paper and washed with 10 μL of 2% uranyl acetate three times. Thirty seconds after the final wash, the grid was blotted and air‐dried for imaging. CryoEM grid was prepared by plunge‐freezing with an FEI Mark IV Vitrobot (Thermo Fisher). Three μL of the sample were applied onto a glow‐discharged thin continuous carbon film‐coated lacey grid. After incubation in the chamber of Vitrobot at 8°C and 100% humidity for 1 min, the grid was blotted for 15 s and plunge‐frozen in liquid ethane. Images for both negative staining and cryogenic grids were obtained with a 200 kV FEI TF20 electron microscopy.

### 4.11 Egress and Invasion Assays

Merozoite egress was analyzed by measuring the egression rate in the mature schizonts. Briefly, the highly synchronized parasites at the mature schizont stage were purified by a 40%/70% Percoll‐sorbitol gradient [75]. The purified schizonts were put back into culture by adding new red blood cells and culture medium, and the egress events were observed under live microscopy for at least 6–8 h. Merozoite invasion was analyzed by the established method [86, 87, 88].

### 4.12 Cell Fractionation and Heme Assays

Heme in hemoglobin (Hb), free heme, and hemozoin (Hz) in the parasites was analyzed by the established methods [49, 50]. Briefly, 5 × 10^8^ highly synchronized iRBCs at late trophozoite stages were lysed by saponin to harvest parasites, followed by hypotonic lysis and centrifugation. Hb was measured in the supernatant after SDS treatment and addition of pyridine. Free heme was obtained by further treatment of the pellet with SDS and pyridine. The undissolved Hz was solubilized in NaOH, followed by neutralization with HCl. Hz was then measured after adding pyridine.

### 4.13 Hemozoin Content Assays

The amount of hemozoin in iRBCs was estimated as previously described [51, 52]. Briefly, 1 × 10^9^ highly synchronized iRBCs at late trophozoite stages were lysed by ice‐cold distilled water. After centrifugation at 4000 rpm at 4°C for 30 min, the pellet was washed with ice‐cold distilled water, dissolved in 1 mL of 0.1 M NaOH, and incubated at 50°C for 10 min. The concentration of hemozoin in the supernatant was determined by measuring the absorbance at 400 nm.

### 4.14 Hemoglobin Content by Flow Cytometry After Dextran Incorporation and RBC Reseal

Hemoglobin inside parasites was analyzed using an established method [89]. First, the resealed RBCs were prepared in the presence of 50 μM fluorescein‐dextran (anionic, 10 kDa; life‐tech). Mature schizonts were added to the resealed RBCs. The green fluorescence signals in the parasite at the trophozoite stage (30 hpi) were examined by flow cytometric analysis after parasites were released from iRBCs by saponin lysis.

### 4.15 Statistical Analysis

Data is represented as mean ± standard deviation from three replicates. T‐tests were used for statistical comparison between growth phenotypes before and after *Pf*CHC KD. *p* < 0.05 or less was taken as statistically significant

## Funding

This work was supported by National Institute of Allergy and Infectious Diseases (NIAID), National Institutes of Health, R01AI128940, U19AI 089672, R21AI173856. Morsani College of Medicine.

## Ethics Statement

The manuscript does not include data or a description of human or animal patients.

## Conflicts of Interest

The authors declare no conflicts of interest.

## Supporting information


**Data S1:** Supporting Figures.

**Table S1A:** IPs from PfCHC::GFP parasite line by GFP‐trap
**Table S1B:** SAINT analysis of IPs of PfCHC‐GFP
**Table S1C:** Published IPs from PfCHC‐2xFKBP‐GFP parasites by anti‐GFP magnetic beads
**Table S1D:** Published PfCHC BioID pulldown
**Table S1E:** Overlaps among different PfCHC IPs.

**Table S2A:** IPs from PfCLC::GFP parasite line by GFP‐trap
**Table S2B:** SAINT analysis of IPs of PfCLC‐GFP
**Table S2C:** Published K13 BioID IPs
**Table S2D:** Shared proteins between IPs from PfCLC::GFP GFP‐trap and K13 BioID

**Table S3A:** IPs from PfAP1‐γ::GFP parasite line
**Table S3B:** SAINT analysis of IPs of PfAP1‐γ‐GFP
**Table S3C:** Shared proteins between IPs from PfAP1 γ::GFP GFP‐trap and K13 BioID

**Table S4:** Proteome of purified CCVs from malaria parasite P. falciparum.

**Table S5:** Primers used for GFP/APEX‐tag or TetR‐DOZI KD

**Video S1:** Dextran‐containing structure in the PfCHC knockdown parasites. A representative three‐dimensional (3D) reconstructed image of a PfCHC knockdown parasite‐infected red blood cell is shown from different angles by this video. A dextran (green)‐containing structure, which is surrendered by the parasite membrane (red, stained by Bodipy‐TR‐C5_ceramide), is localized at parasite periphery. This dextran‐containing structure displays as a long tubular‐like morphology.

**Video S2:** Dextran‐containing structure in the wildtype parasites. This video shows a representative 3D image of a wildtype parasite‐infected red blood cell. A small bubble‐like dextran (green)‐containing structure, which is surrendered by the parasite membrane (red), is localized at parasite periphery.

## Data Availability

All data was made publicly available on common data repositories. The mass spectrometry data have been deposited to the ProteomeXchange Consortium via the PRIDE [90] partner repository with the dataset identifier PXD031805 and 10.6019/PXD031805.
