# Supplementary material for: Clathrins Are Involved in the Endocytosis of Host Cytosol in the Malaria Parasite
Source: Traffic. 2025 Dec 26;27(1):e70025. doi: 10.1111/tra.70025 (PMC12742282; doi:10.1111/tra.70025)
Supplement: Supplementary file 1 — Data S1: Supporting Figures. [file TRA-27-e70025-s005.pdf]

Supplementary Figures

Clathrins are involved in the endocytosis of host cytosol in the malaria parasite

Jun Miao[1,2]*, Amuza Byaruhanga Lucky[1], Gang Ning[3], Xian Xia[4], Xiaoying Liang[1], Faiza Siddiqui[1], Hui Min[1#], Chengqi Wang[2], Xiaolian Li[1], Z. Hong Zhou[4], Liwang Cui[1,2]*

1. Department of Internal Medicine, Morsani College of Medicine, University of South Florida, Tampa, Florida, USA
2. Center for Global Health and Infectious Diseases Research, College of Public Health, University of South Florida, Tampa, Florida, USA
3. Huck Institute of Life Sciences, The Pennsylvania State University, University Park, PA, USA
4. Department of Microbiology, Immunology & Molecular Genetics, University of California, Los Angeles, CA, USA

*Address Correspondence to Jun Miao: jmiao1@usf.edu and Liwang Cui: liwangcui@usf.edu

# Present address: Department of Immunology, College of Basic Medical Sciences, China Medical University, Shenyang, Liaoning, China

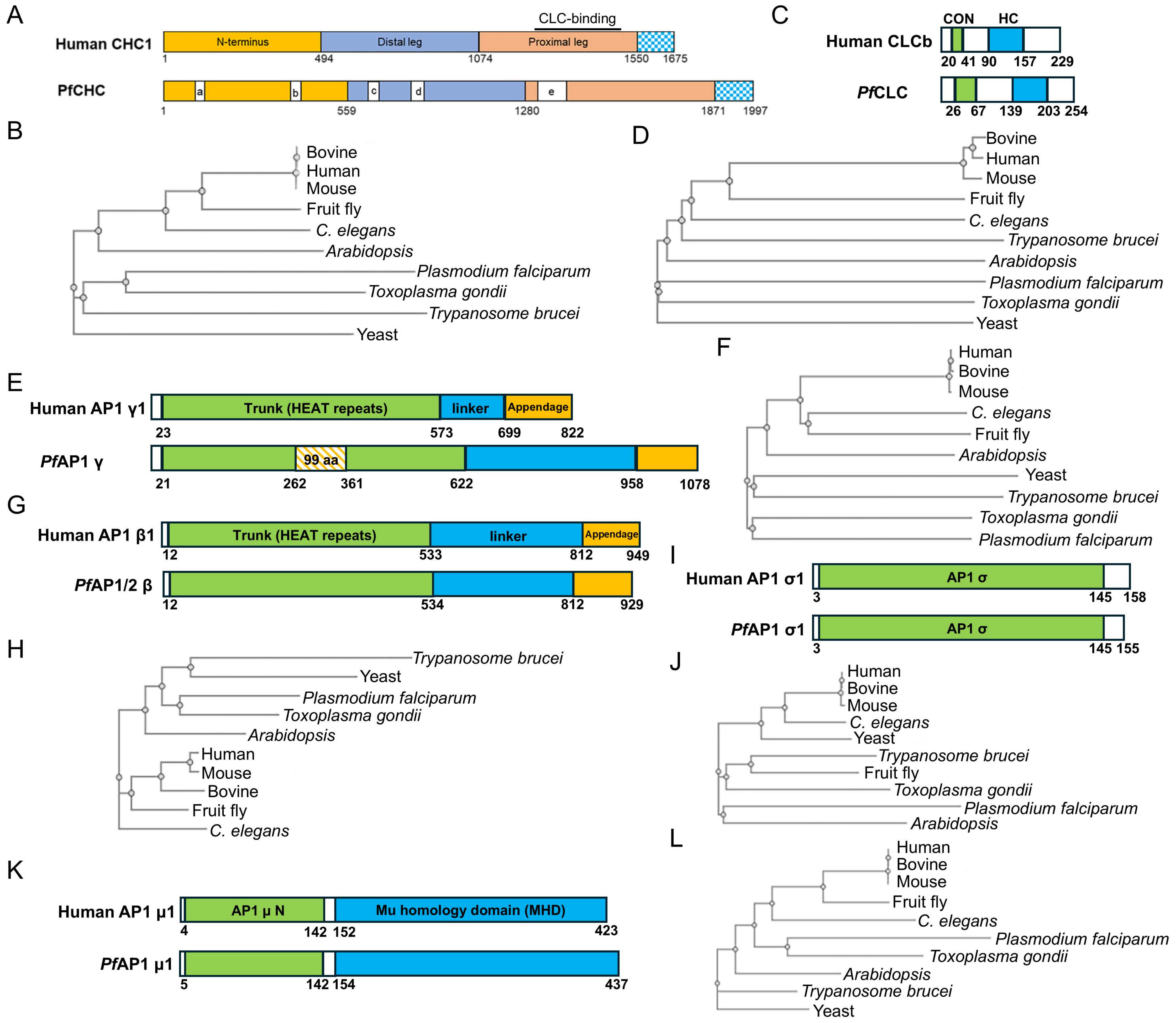

Fig. S1. Comparison of *Pf*CHC, *Pf*CLC, and *Pf*AP1 subunits with their human counterparts. A. *Pf*CHC is 322 amino acids larger than the human CHC1. The predicted four domains in *Pf*CHC match well with those in the human CHC1 except for the Asn-rich sequence insertions (a–e) in *Pf*CHC. The domain that binds to the clathrin light chain (CLC-binding) is indicated. **B**. Neighbor-joining tree of *Pf*CHC with CHCs from model organisms shows the highest similarity of *Pf*CHC to the CHC from *Toxoplasma gondii*. CHCs were retrieved from the GenBank for Bovine (access number P49951), Human (NP_004850), Mouse (Q68FD5), Fruit fly (CAA78507), *C. elegans* (P34574), *Arabidopsis* (AAG50828), Yeast (A36349), *Trypanosome brucei* (XP_822847), and *T. gondii* (A0A7J6KDH2). **C**. *Pf*CLC contains the predicted consensus region (CON) and heavy chain binding region (HC) corresponding to those in the human CLCa and CLCb. **D**. Neighbor-Joining tree shows the phylogeny of *Pf*CLC with CLCs from model organisms, indicating its highest similarity to *T. gondii* CLC. CLCs were retrieved from GenBank for Bovine CLCb (P04975), Human CLCb (P09497), Mouse CLCb (Q6IRU5), Fruit fly (Q9VWA1), *C. elegans* (P90961), *Arabidopsis CLC1*(Q9SKU1), Yeast (P17891), *T. brucei* (Q388C8), and *T. gondii* (A0A7J6KAX2). **E**. *Pf*AP1 γ contains the predicted N-terminal Trunk domain, C-terminal Appendage domain, and the unstructured linker, which align well with those in the human AP1 γ1. A 99 aa insertion in *Pf*AP1 γ is indicated. **F**. Neighbor-Joining tree shows the phylogenetic relationship of *Pf*AP1 γ with AP1 γ subunits from model organisms. AP1 γ subunits were retrieved from GenBank for Bovine (A0AAA9TSD9), Human (O43747), Mouse (P22892), Fruit fly (Q7KVR8), *C. elegans* (Q8WQB3), *Arabidopsis* (Q84K16), Yeast (Q12028), *T. brucei* (Q580A4), and *T. gondii* (A0A7J6JXR2). **G.** *Pf*AP1/2 β contains the predicted N-terminal Trunk domain, C-terminal Appendage domain, and the unstructured linker match well with those in the human AP1 β1. **H.** Neighbor-Joining tree shows the phylogenetic analysis of *Pf*AP1/2 β with AP1 β1 from model organisms indicating its highest similarity to the AP β from *T. gondii***.** AP1 β were retrieved from GenBank for Bovine (access number Q08DS7), Human (Q10567), Mouse (O35643), Fruit fly (Q24253), *C. elegans* (Q9N4F3), *Arabidopsis* (F4JNZ8), Yeast (P36000), *T. brucei* (Q38A51), and *T. gondii* (A0A7J6K8R4). **I.** *Pf*AP1 μ1 contains the predicted AP1 μ N-terminal domain and Mu homology domain (MHD) matching well with those in the human AP1 μ1. **J.** Neighbor-Joining tree shows the phylogenetic analysis of *Pf*AP1 μ1 with AP1 μ1s from model organisms indicating its highest similarity to *T. gondii* AP1 μ1. AP1 μ1s were retrieved from GenBank for Bovine (Q2KJ81), Human (Q9BXS5), Mouse (P35585), Fruit fly (A0A6P8KN78), *C. elegans* (P35602), *Arabidopsis* (Q9SAC9), Yeast (Q00776), *T. brucei* (Q388C8), and *T. gondii* (A0A7J6KAX2). **K.** *Pf*AP1 σ1 contains the predicted AP1 σ domain matching well with those in the human AP1 σ1. **L.** Neighbor-Joining tree shows the phylogenetic analysis of *Pf*AP1 σ1 with AP1 σ1 subunits from model organisms. AP1 σ1 subunits were retrieved from GenBank for Bovine (Q1JQ98), Human (P61966), Mouse (P61967), Fruit fly (B3P034), *C. elegans* (O16369), *Arabidopsis* (Q8LEZ8), Yeast (P35181), *T. brucei* (Q57TU5), and *T. gondii* (A0A7J6K495).

```
P.falciparum      ------------------------------------------------------------MSELKEFDEFNFKNYDNSNNNNEKENDEGPSNIFYEENNEVGTSLDKIN---Y------- 50
T.gondii          MDGDFGATGGGFGDMADFTSSPGLSLNAPVLSADASSPFSRSPRHEQRQPQLSSGSGGYEDVSSSVYSQNDYGCLNGVRS------SPQDDDAFFAEEPPVNDSFLEEN-DVFAMGRENR 113
Yeast             -------------------------------------------------------------------MSEKFPPLEDQNIDFTPNDKKDDDTDFLKREAEILGDEFKTEQDDI------- 46
Arabidopsis       ------------------------------------------------------------------MATFDDGDFPAQTH-----------------SPSEHEDFGGYD--NF------- 28
T. brucei         -------------------------------------------------------------------------------------------MDFLNESQTGQD-IN-QN--SF------- 18
C.elegans         --------------------------------------------------------------------------------------MSDPVADFLAREQNLFADFDGAP--PA------- 25
Fruit fly         ---------------------------------------------------------------------MDFGD------DFAAKEDVDPAAEFLAREQSALGDLEAEI--TG------- 36
Mouse             -------------------------------------------------------------------MAEDFGFFSSSESGAPEAAEEDPAAAFLAQQESEIAGIENDP--GF------- 44
Bovine            -------------------------------------------------------------------MADDFGFFSSSESGAPEAAEEDPAAAFLAQQESEIAGIENDE--GF------- 44
Human             -------------------------------------------------------------------MADDFGFFSSSESGAPEAAEEDPAAAFLAQQESEIAGIENDE--GF------- 44

P.falciparum      ------MNPIEN------VDYNNMNMSSNNFYNELDKRESS---SSLLYNNNNNNNM--------NNINNNIHKKYNGSDIKNNDSFYKSNLSSPIKENRMSFENSYETFF-------EK 140
T.gondii          EVSDGYSPPSLQ----------TGSDATQKRDTSFDDRVGPNAQANGVYGLPQDSPLGEATH-----EEP---PK-----------------SAPG-----Q----------QAPRMASG 183
Yeast             --LETEASPAKDDDEIRDFEEQFPDINSANGAVSSDQ----------------------------------------------NGSATVSSGNDNG-----EADDDFSTFE--GANQSTE 111
Arabidopsis       --SEAQQPPTQH---------QSGGFSSFNGDPASPN----------GYGFGA--------SSPNHDFSSPFESSV-------ND-ANGNGGGSGG-----D-----AIFASDGPILPDP 101
T. brucei         --NGNAGPALPN------S---------EEGDNTISSEA--------PSGVPAMPQASEAAVIPQTPQTPQIPPQT-------A------------------------------------ 70
C.elegans         --AAAANPDAPE------ADAPAPALDDDFGDLQIAGDEPPPVVHPTDSGVDLDGLVDDNAAAPA-IVVPAVEPMV-------NGNHSASSGGSKG-----P-----SPIL-------S- 111
Fruit fly         --GSASAPPAAS---------TDEGLGELLGGTASEGDL---LSAGGTGGLESS-----------------------------TGSFEVIGGESNE-----P-----VGIS-------GP 96
Mouse             --GAPAASQVAS---------AQPGLASGAG---SEDMS---T--------TVN-----------------------------GDVFQEANGPADG-----Y-----AAIA-------QA 93
Bovine            --GAPAGSQGGL---------AQPGPASGA----SEDMG---A--------TVN-----------------------------GDVFQEANGPADG-----Y-----AAIA-------QA 92
Human             --GAPAGSHAAP---------AQPGPTSGAG---SEDMG---T--------TVN-----------------------------GDVFQEANGPADG-----Y-----AAIA-------QA 93

P.falciparum      ESDISDTEVSDVW-EK---ERLQRIKERKEYEEKEKKEIKKKAAQDLKKWYEEIAIVIEEKKKLSNQKLSE-------------------DKKKEQNMDNKTWLKVSQYLDME------- 230
T.gondii          SVEAEIDEQARQAAEAMQEQLRQEVEGRRREEEALKREQRELAQEELKDFYERRKQMIERRSKANQAKENE-------------------TPNGFADNREGSWTRVIQLIDADDRVRSRA 284
Yeast             SVKEDRSEVVDQW-KQ---RRAVEIHEKDLKDEELKKELQDEAIKHIDDFYDSYNKKKEQQLEDAAKEAEAFLKKRD-------------EF---FGQDNTTWDRALQLINQDDAD---- 207
Arabidopsis       NEMREEGFQRREW-RR---LNTIHLEEKEKKEKEMRNQIITEAEDFKKAFYEKRDKTIETNKTDNREKEKLYWANQ--------------EKFHKEV-DKHYWKAIAELIPREVPNIEKK 202
T. brucei         APVQQSSATIAVC-QAAKKGIDARTAEIDAKSREKERQLTEAAQAYLKEQNKMREEKIKEVKAKHVK------------------EQEVQEKQKPCSDENAVWVNVGKMVAFN------- 164
C.elegans         TVPRIEAEKIRLW-KA---QQEQLLSKKDEAEEKKKIELRANAKKELEEWYKQREKTLQLSHDENLKNEKS-----------------NQELFAKQQDGDAQWETVNKLVDQ-------- 202
Fruit fly         PPSREEPEKIRKW-RE---EQKQRLEEKDIEEERKKEELRQQSKKELDDWLRQIGESISKTKLASRNAEKQ-----------------AATLENGTIEPGTEWERIAKLCDFN------- 188
Mouse             DRLTQEPESIRKW-RE---EQKKRLQELDAASKVTEQEWREKAKKDLEEWNQRQSEQVEKNKINNRIADKAFYQQPDADTIGYVASEEAFVKESKEETPGTEWEKVAQLCDFN------- 202
Bovine            DRLTQEPESIRKW-RE---EQRKRLQELDAASKVMEQEWREKAKKDLEEWNQRQSEQVEKNKINNRIADKAFYQQPDADIIGYVASEEAFVKESKEETPGTEWEKVAQLCDFN------- 201
Human             DRLTQEPESIRKW-RE---EQRKRLQELDAASKVTEQEWREKAKKDLEEWNQRQSEQVEKNKINNRIADKAFYQQPDADIIGYVASEEAFVKESKEETPGTEWEKVAQLCDFN------- 202

P.falciparum      ------------------KGEYFKENSRMKQVLLKLIQKESS------------------------------------------------------------------------------ 254
T.gondii          -GSSAA-----GAATSASATASSKDRCKMRELLVELSAQEKPGESN-------------------------------------------------------------------------- 324
Yeast             -------------------IIGGRDRSKLKEILLRLKGNAKAPGA--------------------------------------------------------------------------- 233
Arabidopsis       RGKKDPDKKPSVNVIQGPKPGKPTDLGRMRQIFLKLKTNPPPHMMPPPPPAKDAKDGKDAKDGKDAKTGKDGKDAKGGKDAKDLKDGKPADPKVTEEKRPSPAKDASVETAKPDAAASGE 322
T. brucei         -----------------KVNKYSKNTERMRSILGKLSQSGSG------------------------------------------------------------------------------ 189
C.elegans         -----------------QKSKSGKDLSRLKTLLAGLKHAGK------------------------------------------------------------------------------- 226
Fruit fly         ---------P-------KVNKAGKDVSRMRSIYLHLKQNPIQVQKST------------------------------------------------------------------------- 219
Mouse             ---------P-------KSSKQCKDVSRLRSVLMSLKQTPLSR----------------------------------------------------------------------------- 229
Bovine            ---------P-------KSSKQCKDVSRLRSVLMSLKQTPLSR----------------------------------------------------------------------------- 228
Human             ---------P-------KSSKQCKDVSRLRSVLMSLKQTPLSR----------------------------------------------------------------------------- 229

P.falciparum      ---------------- 254
T.gondii          ---------------- 324
Yeast             ---------------- 233
Arabidopsis       GEKPVAVTEAEGTKAE 338
T. brucei         ---------------- 189
C.elegans         ---------------- 226
Fruit fly         ---------------- 219
Mouse             ---------------- 229
Bovine            ---------------- 228
Human             ---------------- 229
```

Fig. S2. Amino acid sequence alignment of clathrin light chain (CLC).
P. falciparum PfCLC was aligned with CLCs retrieved from Bovine CLCb (P04975), Human CLCb (P09497), Mouse CLCb (Q6IRU5), Fruit fly (Q9VWA1), *C. elegans* (P90961), *Arabidopsis CLC1*(Q9SKU1), Yeast (P17891), *T. brucei* (Q388C8), and *T. gondii* (A0A7J6KAX2). The acidic patch EED in human/mouse/bovine CLC is relatively conserved in *Pf*CLC (DEG). The residues W105 and W127 in human/mouse/bovine CLC critical for binding CHC are relatively conserved in *Pf*CLC (W153 and I175).

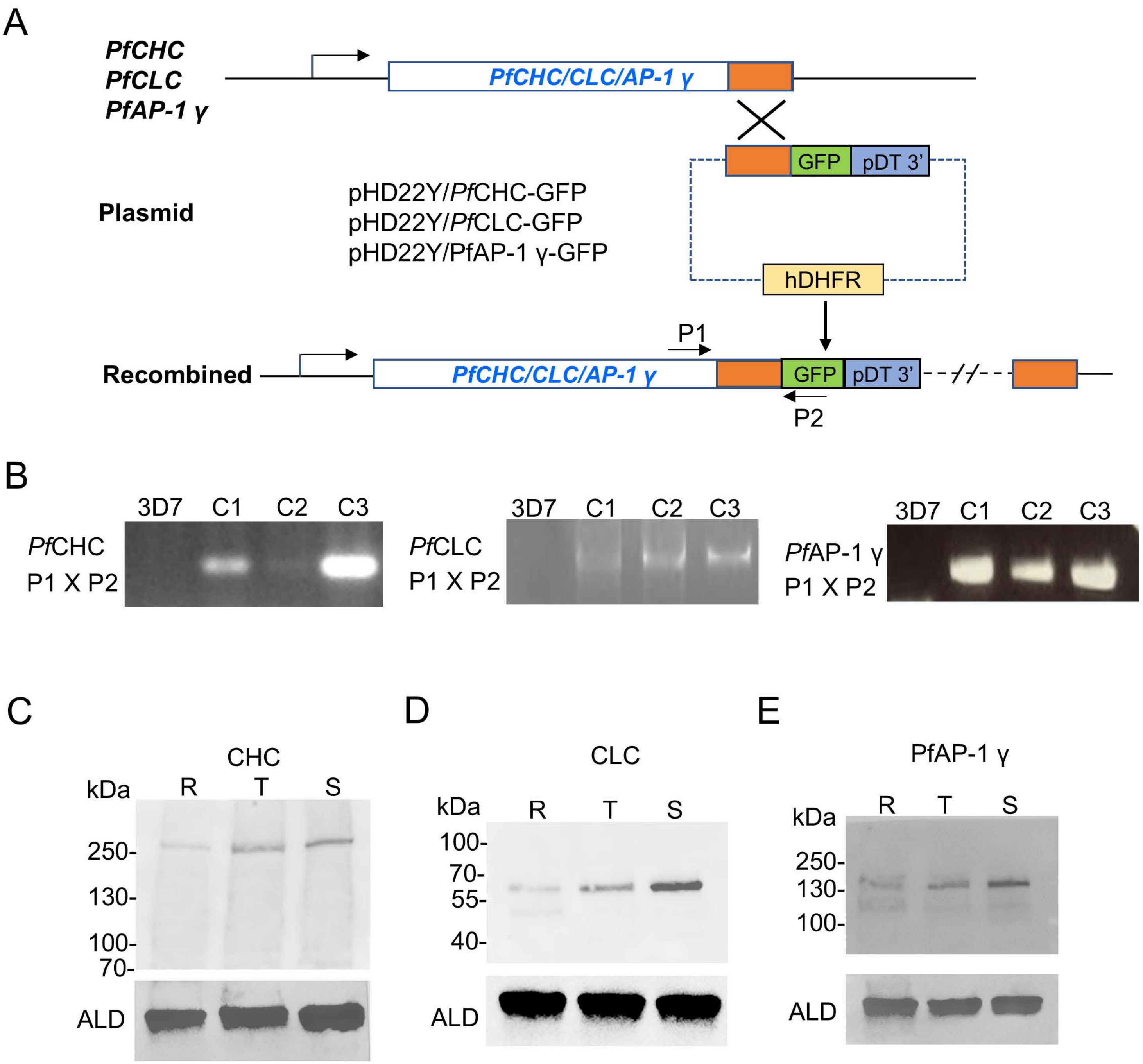

Fig. S3. GFP tagging of *PfCHC*, *PfCLC*, and *PfAP-1 γ*.
A. Diagram of the GFP tagging strategy for *PfCHC, PfCLC,*and *PfAP-1 γ* shows the integration event resulting from single cross-over recombination. P1 and P2 are two primers for PCR diagnostic analysis of integration. **B**. Diagnostic PCR showing the positive clones from *PfCHC, PfCLC,*and *PfAP-1 γ* tagging, respectively. For each gene tagging, 3 positive clones were tested. A Wild-type 3D7 parasite line was used as the control. **C- E**. Western blot analysis of *Pf*CHC-GFP (**C**), *Pf*CLC-GFP (**D**), and *Pf*AP-1 γ (**E**) expression in *Pf*CHC::GFP clone (C1), *Pf*CLC::GFP clone (C1), and *Pf*AP-1 γ::GFP (C1), respectively, with the anti-GFP antibodies. Protein extracts of the synchronized ring (R), trophozoite (T), and schizont (S) stages were separated in an 8% SDS-PAGE gel. Each lane had ~10 ug of protein extracts. Protein loading was monitored by the anti-aldolase antibodies (ALD).

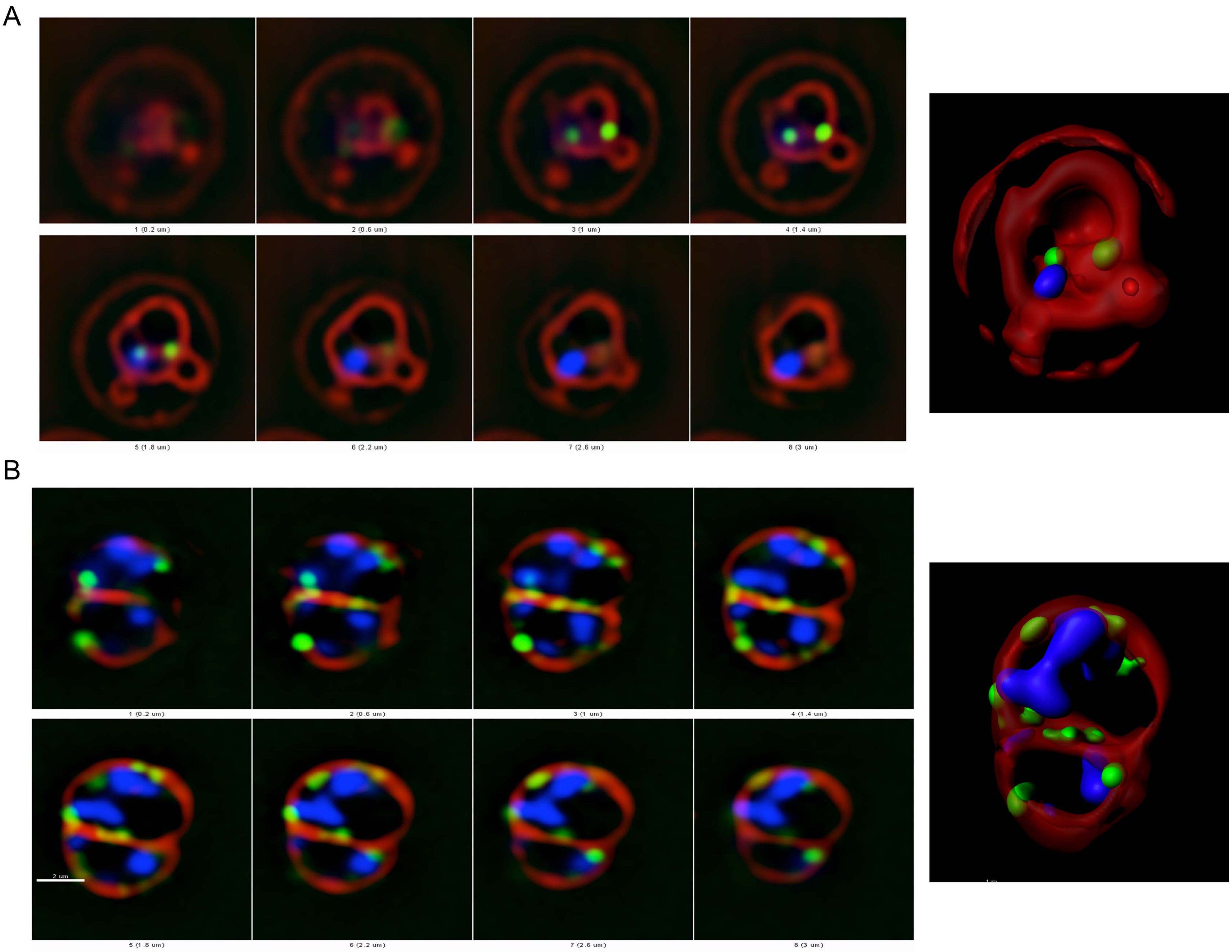

Fig. S4. *Pf*CHC localization by SIM. A and B SIM showed 10 consecutive layers of two RBC infected by *Pf*CHC-GFP parasites at late stage of the ring (**A**) and trophozoite (**B**) stage and their reconstructed 3D images (left) with Bodipy membrane staining (red) and nuclear staining (blue).

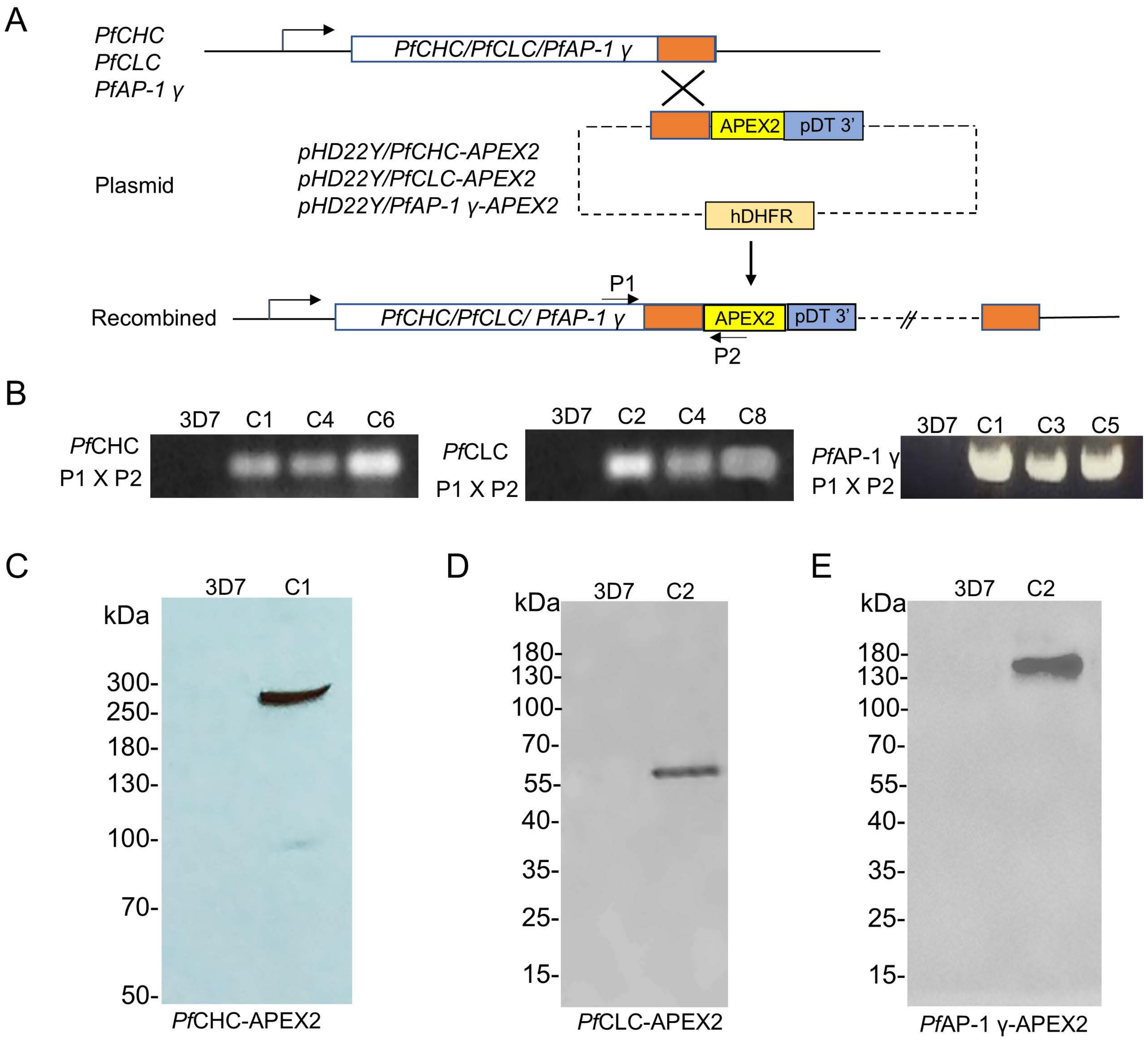

Fig. S5. Tagging *Pf*CHC, *Pf*CLC, and *Pf*AP-1 γ with APEX2. **A**. A diagram shows the APEX2 tagging strategy. P1 and P2 are two primers for PCR diagnostic analysis of integration. **B.** Diagnostic PCR for the positive clones of *Pf*CHC::APEX2, *Pf*CLC::APEX2, and *Pf*AP-1 γ::APEX2 compared with wildtype parasite 3D7 as control. **C-E.** Western blot revealed the expression of *Pf*CHC-APEX2 in *Pf*CHC::APEX2 clone 1 (C1) (**C**), *Pf*CLC-APEX2 in PfCLC::APEX2 clone 2 (C2) (**D**), and *Pf*AP-1 γ-APEX2 clone 2 (**E**) with the anti-APEX2 antibodies. The predicted size of *Pf*CHC-APEX2, *Pf*CLC-APEX2, and *Pf*AP-1 γ-APEX2 are ~260, ~58, and ~ 150 kDa, respectively. 3D7 parasites were used as negative controls.

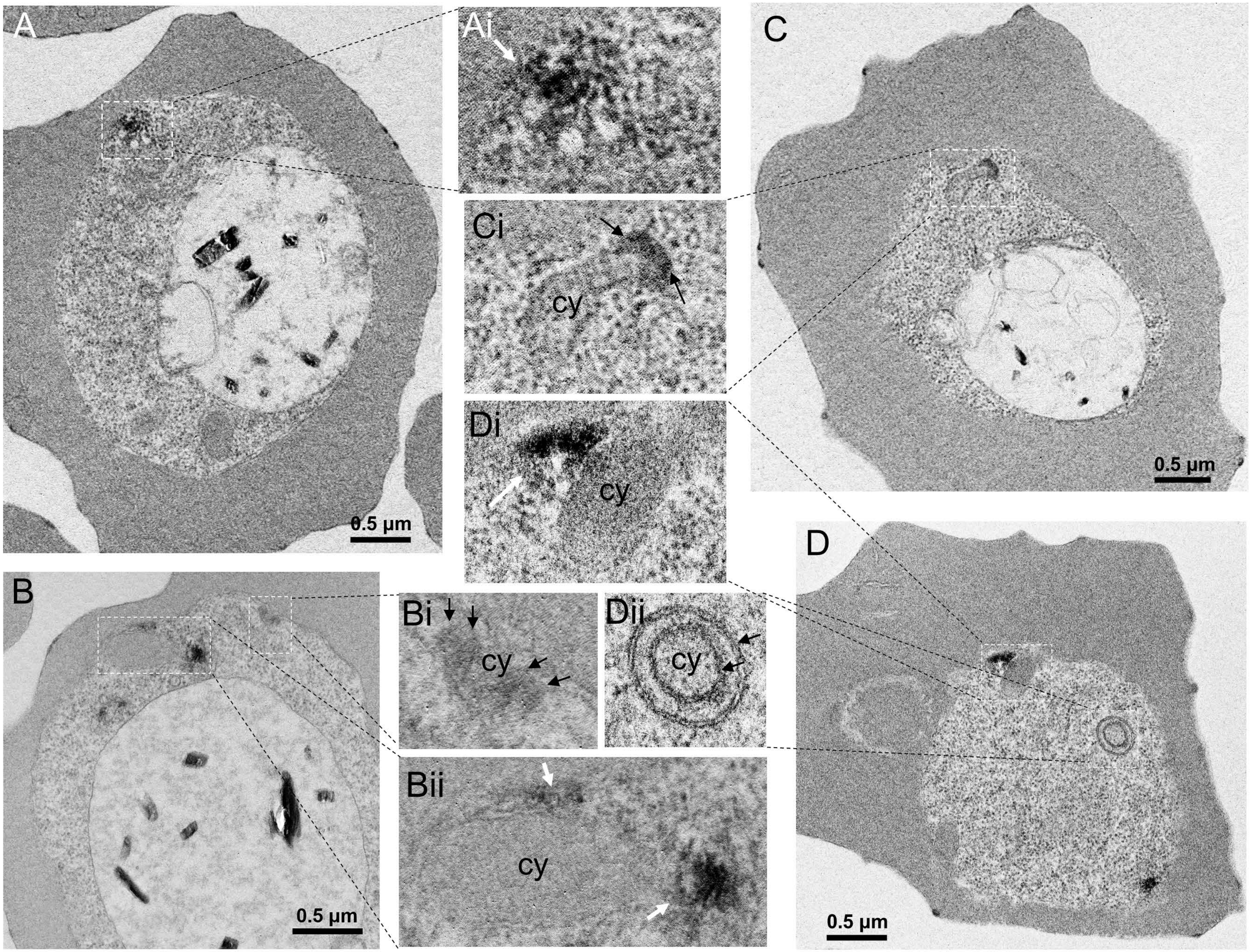

Fig. S6. *Pf*CLC localization in *Pf*CLC::APEX2 parasites by APEX2-based EM.
A. A representative image shows that electron-dense materials are localized in vesicle-like structures in the parasite cytosol (white arrow in the insert **Bi**). **B-D**. Three EM images show that electron-dense materials are localized at cytostome necks (black arrows in the inserts **Bi**, **Ci**, and **Dii**). Two cytostomes in **B** and **C** were vertically sectioned (inserts **Bi** and **Ci**) while a cytostome in **D** was horizontally cut (insert **Dii**). Electron-dense materials also coat vesicle-like structures in near cytostome in **B** and **D** (white arrows in the inserts **Bii** and **Di**). cy: cytostome.

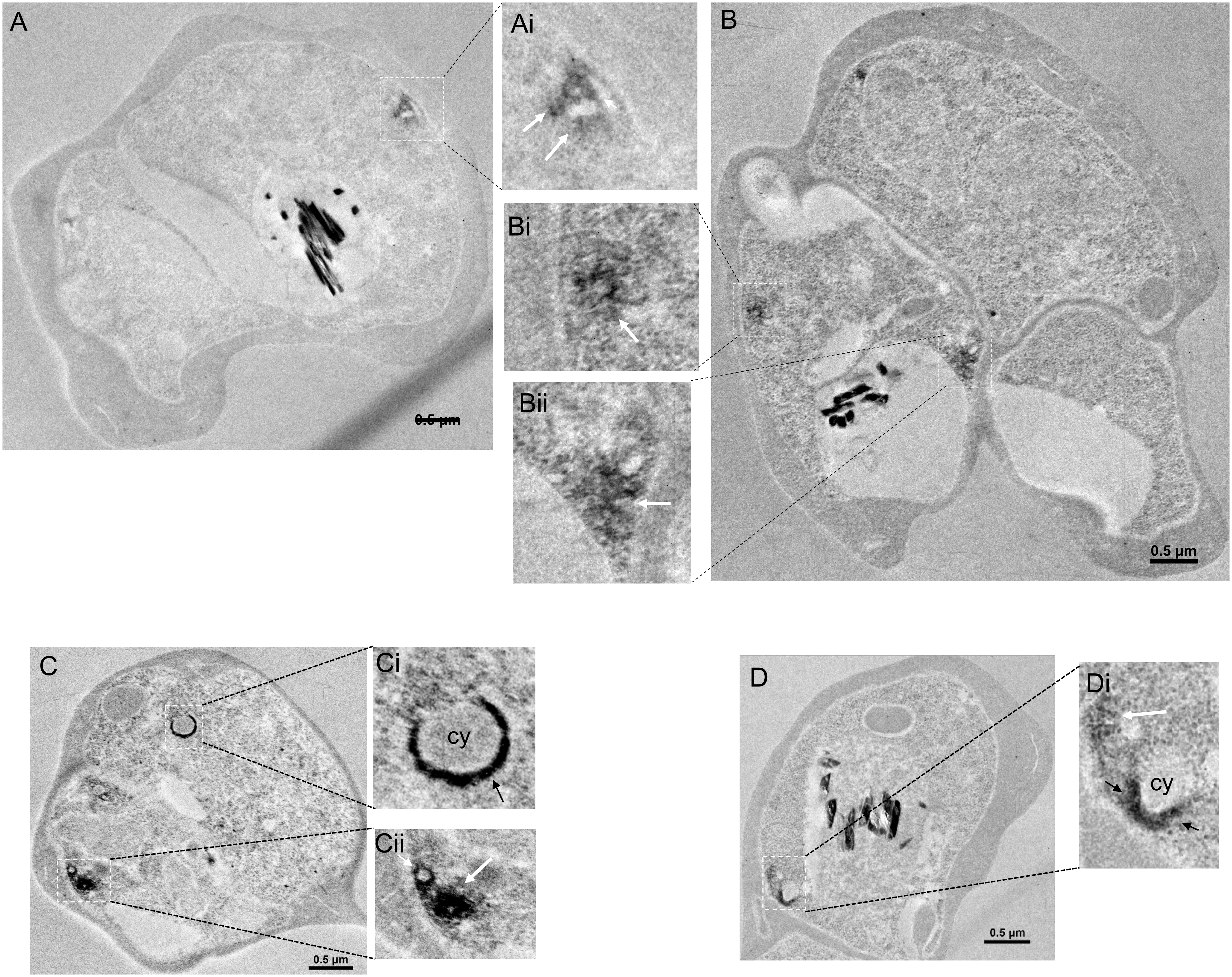

Fig. S7. *Pf*AP-1 γ localization in *Pf*AP-1 γ::APEX2 parasites by APEX2-based EM.
A-C. Three representative images show that electron-dense materials are localized in vesicle-like structures at the parasite periphery (white arrows in the inserts **Ai**, **Bi**, **Bii**, and **Cii**). **C A** representative EM image show that electron-dense materials are localized at cytostome necks, which were horizontally sectioned (black arrows in the inserts **Ci)**. **D**. A representative EM image reveals that electron-dense materials are localized at the cytostome neck (black arrows in the insert **Di**) and the vesicle-like structures near the cytostome (white arrow in the insert **Di**). cy: cytostome.

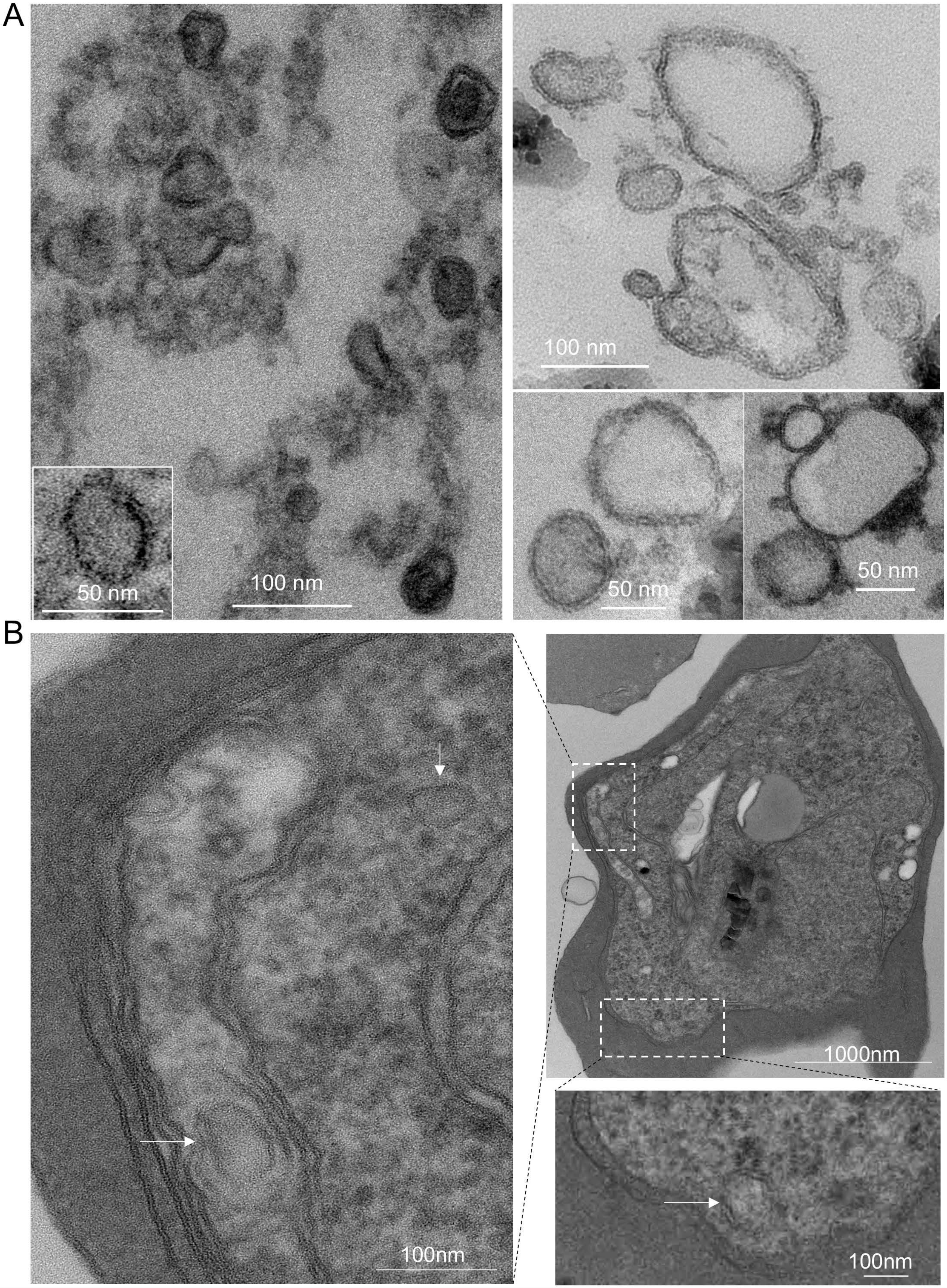

Fig. S8. EM analyses of the purified CCVs and CCV-like structures in the parasites. A. Examples of CCVs purified from *Pf*CHC::GFP parasites using anti-GFP magnetic beads. Purified materials were embedded in Epoxy resin and the ultra-sections were analyzed by transmission EM (TEM). **B**. A representative image of conventional TEM shows some double-membrane-like vesicles which might be the CCVs in the parasite cytosol.

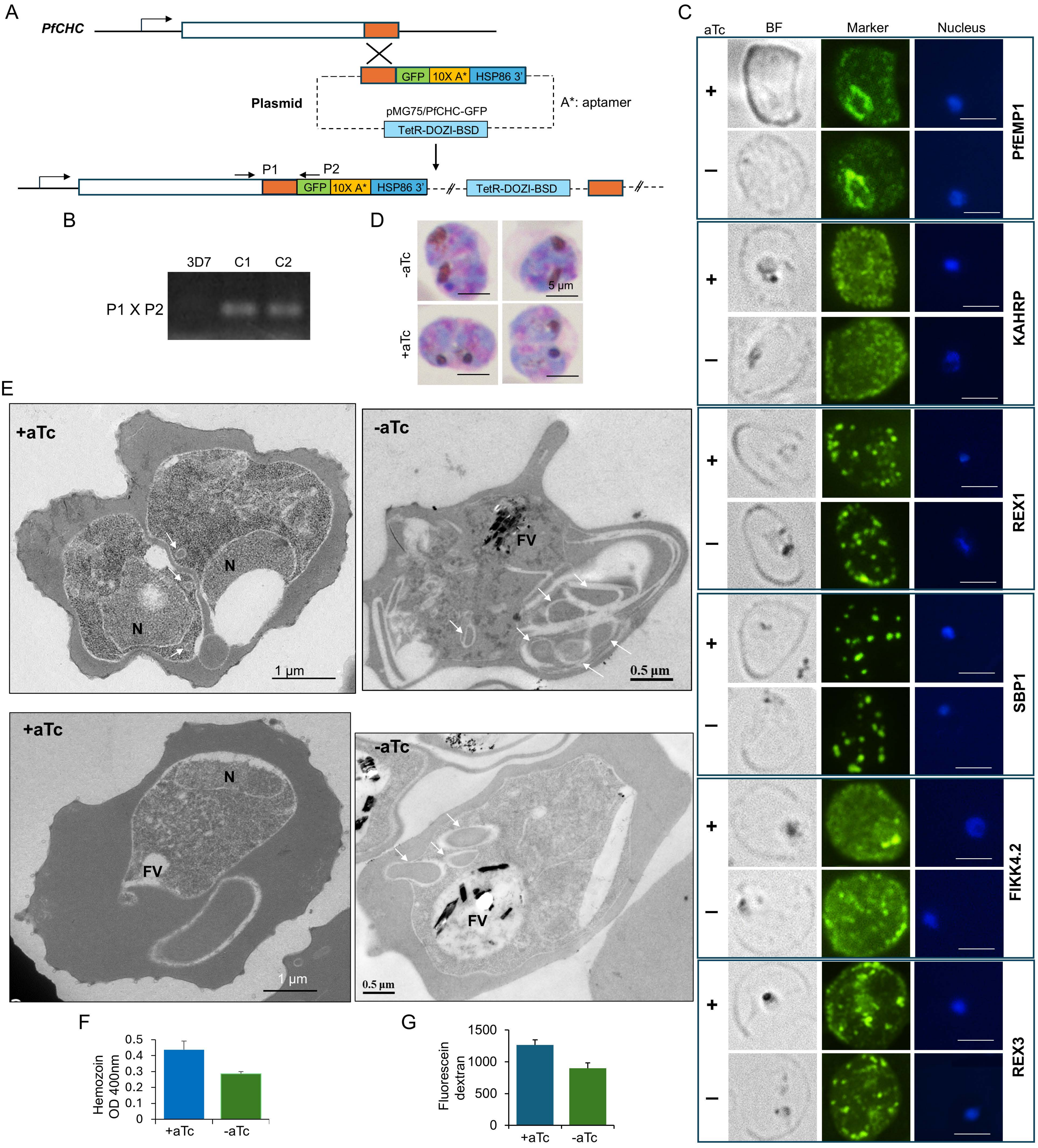

Fig. S9. Knockdown of *Pf*CHC by TetR-DOZI. A. A diagram shows the integration of TetR-DOZI by a single cross-over. *Pf*CHC will be tagged with GFP and 10 repeats of Aptamer will be inserted at the 3' UTR region. TetR-DOZI expression cassette from the plasmid will also be integrated. P1 and P2 are two primers for PCR diagnostic analysis of integration. **B**. Diagnostic PCR for the positive clones of TetR-CHC::GFP. A wild-type parasite 3D7 as a control. **C**. IFA with specific antibodies showed the export of four PNEP (PfEMP1, SBP1, REX1, and REX3), and two PEXEL-containing protein (KAHRP and FIKK4.2) was not disturbed after PfCHC KD for 48h (-aTc) (aTc was withdrawn at early trophozoite stage for 48h) compared to their controls (+aTc). The parasite nuclei were stained by DAPI. The size of the scale bar is 5 µm. BF: bright field. **D**. Giemsa images show the size and color of hemozoin segments in between KD (-aTc) and control (+aTc) trophozoites**G**. **E**. Conventional EM revealed the ultrastructures of iRBC before (+aTc) and after (-aTc) *Pf*CHC KD. N: nucleus. FV: food vacuole. The white arrows indicate the normal and abnormal cytostomes before (+aTc) and after (-aTc) *Pf*CHC KD, respectively. **F.** A bar graph shows hemozoin levels in the synchronized parasites at the trophozoite stage before (+aTc) and after (-aTc) PfCHC KD. Hemozoin was measured by the absorbance at 400 nm. **: $P < 0.01$, t-test***. G.*** A bar graph displays the overall levels of fluorescein-dextran uptake from resealed RBCs into the trophozoite-stage parasites before (+aTc) and after (-aTc) PfCHC KD. The green fluorescence signals of fluorescein-dextran were measured by flow cytometry. *: $P < 0.05$, t-test.

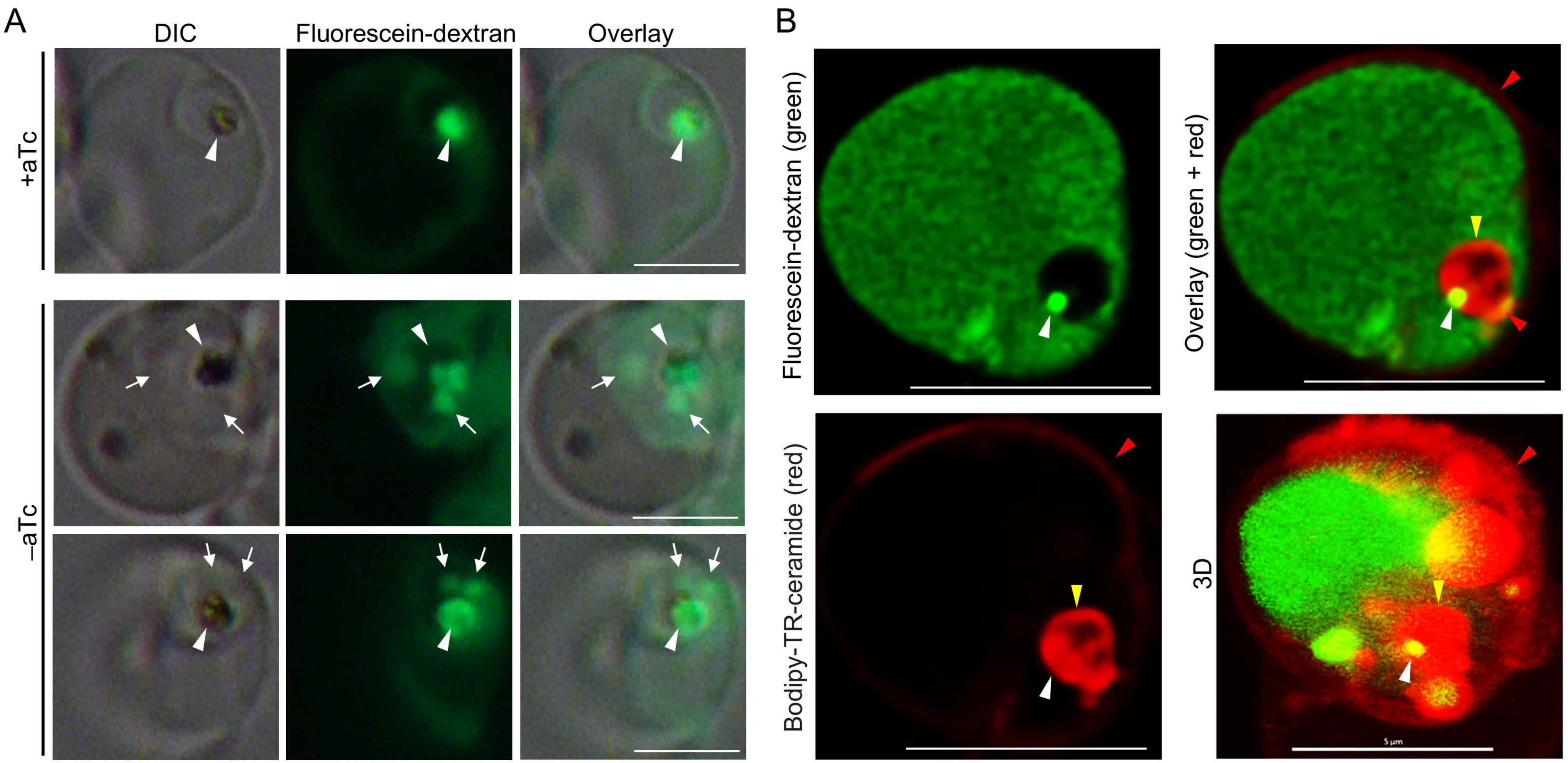

Fig. S10. Dextran-containing structures in the parasites. A. Representative images show the dextran (green)-containing structures in the parasites under fluorescence microscopy. The white arrows indicate the dextran-containing structures in the PfCHC KD parasites (−aTc), whereas no such structure was seen in the wildtype parasites (+aTc). The white arrowheads denote the hemozoin segments, which overlap with strong dextran signals where the food vacuole is located in each parasite. DIC: Differential Interference Contrast. The size bar: 5μm. **B**. Representative super-resolution images display the dextran-containing structures in a wildtype parasite (+aTc) with membrane staining by lipid dye (Bodipy-TR-C5_ceramide). The signals from dextran (green), membrane (red), and their overlay in the parasite-infected red blood cell are shown from a confocal z stack (section), respectively. A three-dimensional (3D) reconstructed image was shown from an angle. Please see supplementary video 2 for 3D images from all angles. The arrowhead indicates a food vacuole-like membrane (red) structure containing dextran (green). A yellow arrowhead indicates the parasite membrane, while a red arrowhead denotes the red blood cell membrane. The size bar: 5μm.

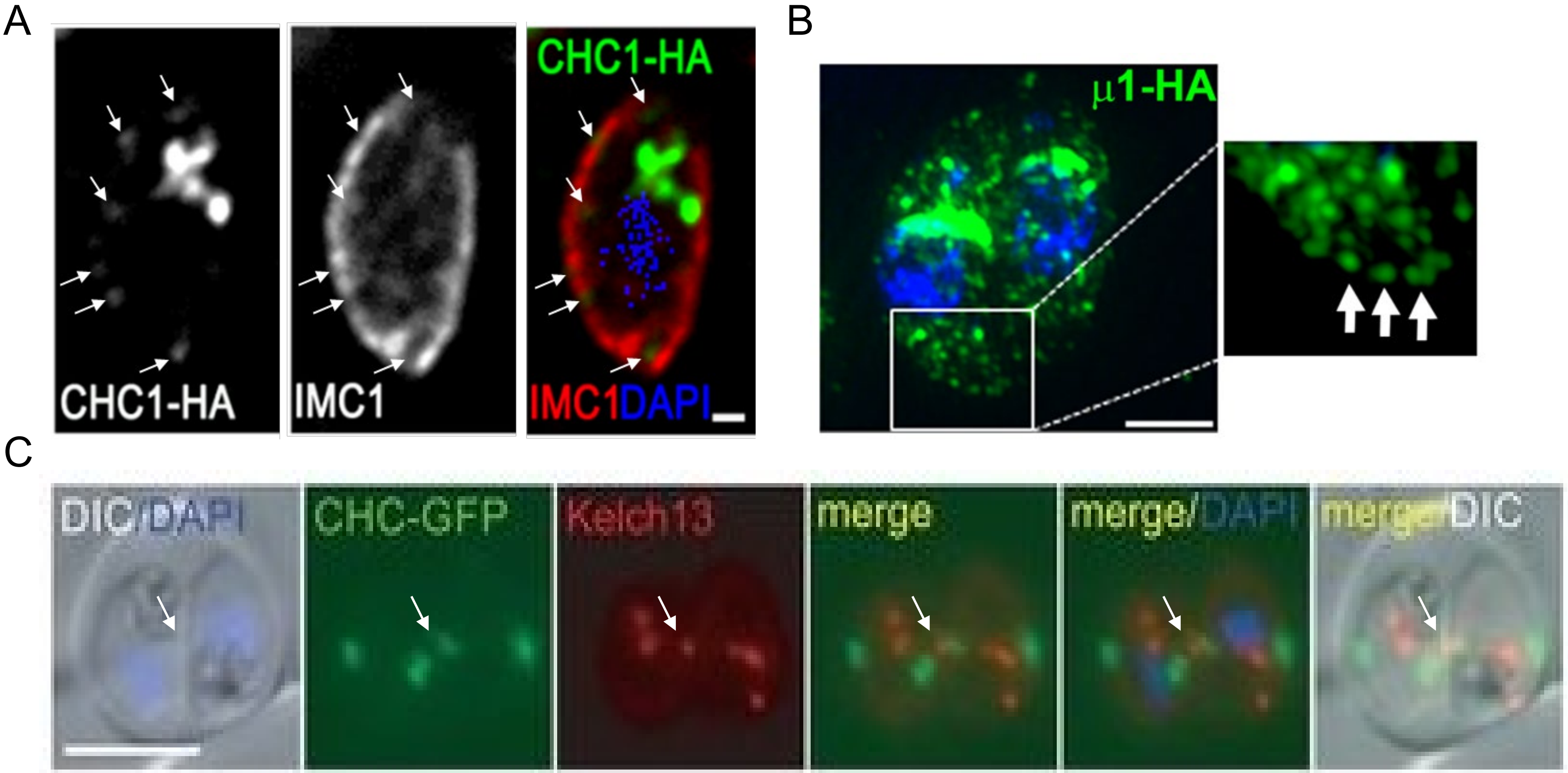

Fig. S11. The published localizations of *Tg*CHC, *Tg*AP-1 µ1, and colocalization between *Pf*CHC and *Pf*K13. **A**. Images were cited from the left panel of Figure 1E of a publication (1). Besides predominantly localized apical to the nucleus, *Tg*CHC was also localized on the parasite plasma membrane (PPM) and at the openings of IMCs (white arrows). Scale bar: 1 µm. **B**. Images were cited from Figure 1D of a paper (2). This SIM image shows that *Tg*AP-1 µ1-HA (green) was localized in vesicles spreading throughout the parasite cytoplasm and in proximity to the PPM (white arrows in the inset). Scale bar: 2 µm. **C**. Images were cited from Figure 2A upper panel of an article (3). A white arrow shows Kelch13 (*Pf*K13) is juxtaposed with *Pf*CHC at the parasite boundary. Scale bar: 5 µm.

References

(1) Pieperhoff MS, Schmitt M, Ferguson DJP, Meissner M. The Role of Clathrin in Post-Golgi Trafficking in *Toxoplasma gondii*. PLOS ONE 2013, 8(10): e77620)

(2) Venugopal K, Werkmeister E, Barois N, Saliou JM, Poncet A, et al. Dual role of the *Toxoplasma gondii* clathrin adaptor AP1 in the sorting of rhoptry and microneme proteins and in parasite division. PLOS Pathogens 2017, 13(4): e1006331

(3) Birnbaum J, Scharf S, Schmidt S, Jonscher E, Hoeijmakers WAM, Flemming S, et al. A Kelch13-defined endocytosis pathway mediates artemisinin resistance in malaria parasites. Science. 2020;367(6473):51-9.
